# Histone Deacetylase 3 Depletion in Osteo/Chondroprogenitor Cells Decreases Bone Density and Increases Marrow Fat

**DOI:** 10.1371/journal.pone.0011492

**Published:** 2010-07-09

**Authors:** David F. Razidlo, Tiffany J. Whitney, Michelle E. Casper, Meghan E. McGee-Lawrence, Bridget A. Stensgard, Xiaodong Li, Frank J. Secreto, Sarah K. Knutson, Scott W. Hiebert, Jennifer J. Westendorf

**Affiliations:** 1 Department of Orthopedic Surgery and Department of Biochemistry and Molecular Biology, Mayo Clinic, Rochester, Minnesota, United States of America; 2 Department of Biochemistry, Vanderbilt University, Nashville, Tennessee, United States of America; Ohio State University, United States of America

## Abstract

Histone deacetylase (Hdac)3 is a nuclear enzyme that contributes to epigenetic programming and is required for embryonic development. To determine the role of Hdac3 in bone formation, we crossed mice harboring loxP sites around exon 7 of *Hdac3* with mice expressing Cre recombinase under the control of the osterix promoter. The resulting *Hdac3* conditional knockout (CKO) mice were runted and had severe deficits in intramembranous and endochondral bone formation. Calvarial bones were significantly thinner and trabecular bone volume in the distal femur was decreased 75% in the *Hdac3* CKO mice due to a substantial reduction in trabecular number. *Hdac3*-CKO mice had fewer osteoblasts and more bone marrow adipocytes as a proportion of tissue area than their wildtype or heterozygous littermates. Bone formation rates were depressed in both the cortical and trabecular regions of *Hdac3* CKO femurs. Microarray analyses revealed that numerous developmental signaling pathways were affected by *Hdac3*-deficiency. Thus, *Hdac3* depletion in osterix-expressing progenitor cells interferes with bone formation and promotes bone marrow adipocyte differentiation. These results demonstrate that Hdac3 inhibition is detrimental to skeletal health.

## Introduction

The skeleton is a dynamic organ composed of more than 200 bones that develop via two distinct processes. Intramembranous ossification occurs when mesenchymal cells condense and differentiate into osteoblasts to form the flat bones of the skull and clavicles. In contrast, the long bones develop via a process of endochondral ossification that involves the recruitment of osteoblasts to a cartilaginous template. Mature osteoblasts secrete collagens and other proteins to form an organic matrix (osteoid) that is mineralized when inorganic salts are brought to the site via the circulation. Osteoblasts are derived from several multipotent progenitors, including mesenchymal cells, neural crest cells and pericytes. Osteoblast development requires the expression of several transcription factors (e.g. Runx2 [1], [2] and osterix [3]) and the coordinated activation of numerous developmental signaling pathways [4]. Cells committed to the osteoblast lineage mature through successive stages of proliferation (pre-osteoblasts), cell cycle exit and production of matrix proteins (osteoblasts), and finally, terminal differentiation into mechanosensory osteocytes or bone lining cells. If progenitor cells do not receive the proper signals for osteogenesis, as in the case of *Runx2*-deficiency, they have an increased propensity to differentiate into adipocytes [5], [6].

Histone deacetylases (Hdacs) are enzymatic components of large multi-protein complexes that remove acetyl groups from lysine side chains of histones and other proteins. They contribute to epigenetic programming and regulation of gene expression during development and throughout life. The human and mouse genomes encode 18 Hdacs that are classified into four groups on the basis of their structure and function [7]. Hdac3 (OMIM 605166) is part of class I, which also includes Hdac1, 2, and 8. Although class I Hdacs are broadly expressed, these predominantly nuclear enzymes have distinct developmental roles [8], [9], [10], [11], [12], [13]. By comparison, class II Hdacs (Hdacs 4, 5, 6, 7, 9, 10) travel between the nuclear and cytoplasmic compartments and have more tissue restricted expression patterns [7], [14], [15], [16], [17]. Hdac3 binds several class II Hdacs [18], [19] and co-repressor proteins, notably nuclear receptor co-repressor (NCoR) and silencing mediator of retinoid and thyroid hormone receptors (SMRT), to form large multi-protein complexes [20], [21], [22]. These multi-protein complexes are recruited to specific DNA sequences and chromatin substrates by transcription factors.

Several Hdacs contribute to skeletal development [7], [23]. Hdac4 is expressed in prehypertrophic chondrocytes [14]. Germline deletion of *Hdac4* caused premature ossification of the developing bones, while Hdac4 overexpression prevented chondrocyte hypertrophy and endochondral ossification [14]. *Hdac6*-deficiency modestly enhanced trabecular bone formation [15]. Recently, *Hdac8* was shown to be essential for neural crest progenitor cell differentiation and skull bone formation [13]. Hdac4 and Hdac6 bind and inhibit the activity of Runx2, a transcription factor essential for osteoblast development [14], [24], [25]. Hdac3 also interacts with Runx2 to repress the expression of osteoblast-specific genes, osteocalcin and bone siaoloprotein, in vitro [24], [26], [27], [28], [29]. Suppression of Hdac3 in immortalized osteoblast cell lines by RNA interference promoted the expression of these genes and other markers of osteoblast maturation [24]. Germline *Hdac3*-deficiency caused embryonic lethality at approximately 9 days post coitum [11], [12], several days before skeletons form in the mouse embryo. In this study, we determined the consequences of *Hdac3* depletion on bone formation by crossing mice with loxP sites flanking exon 7 of *Hdac3* [11] to mice wherein Cre recombinase is expressed from the endogenous *osterix (Osx)* promoter [30]. The data presented here show that *Hdac3* depletion impaired both trabecular and cortical bone properties and promoted bone marrow adipogenesis.

## Results

### Hdac3 Conditional Knockout Mice Have Defects in Intramembranous Bone Formation

**Figure 1:**
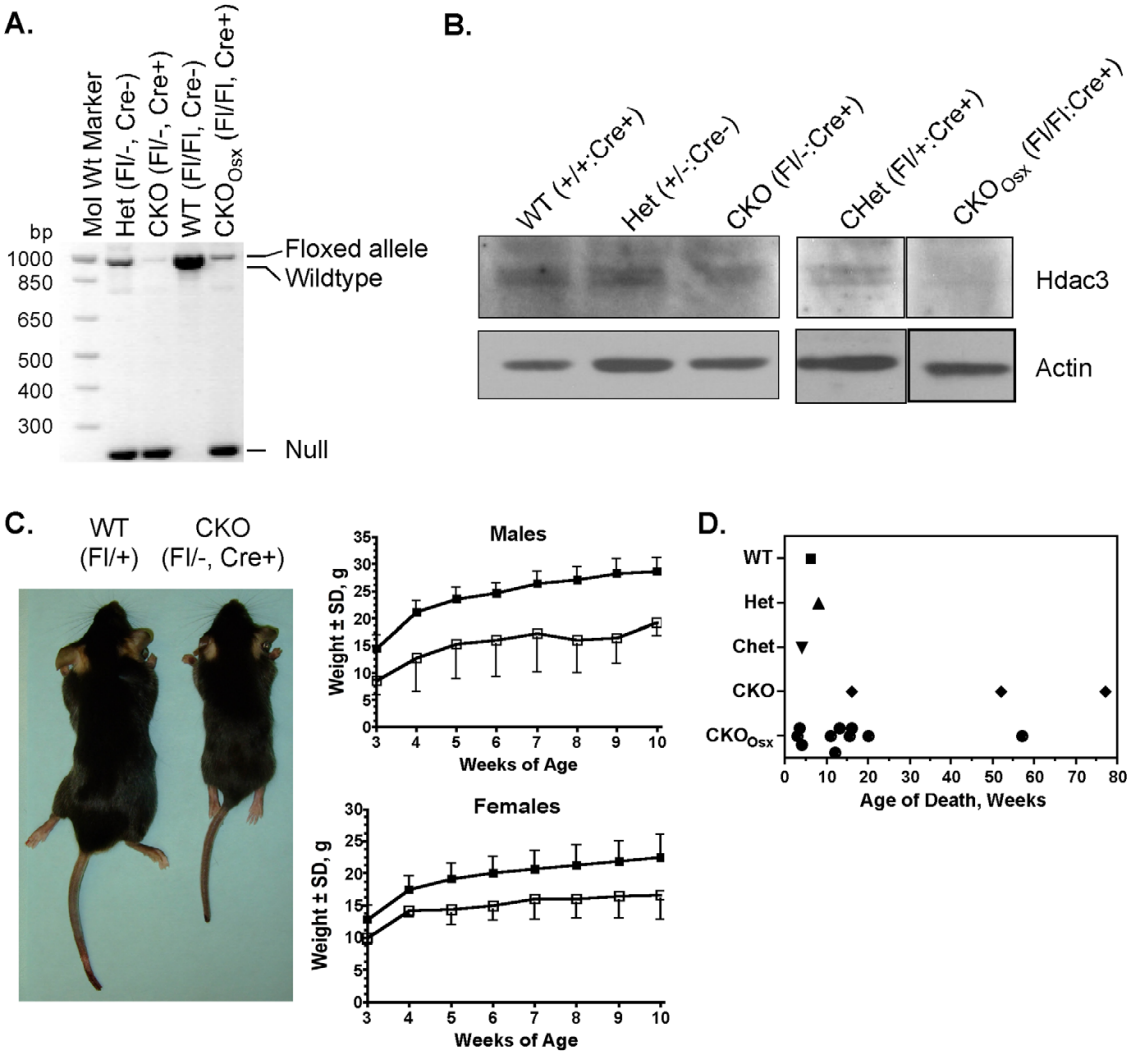
*Hdac3*-deficient mice are smaller and have shorter lifespans. A. PCR amplicons of *Hdac3* alleles. B. Western blot analysis of Hdac3 expression in calvaria. C. Representative 5.5 week-old male wildtype (WT) and *Hdac3* CKO mice are shown in the left panel. Average weekly weights of male and female wildtype (closed squares) and Hdac3 CKO (open squares) mice at and after the time of weaning are presented in the graphs. D. *Hdac3*-CKO and -CKO_Osx_ mice had shorter lifespans than wildtype and heterozygous littermates. Each symbol represents a mouse that died from natural causes.

The effects of *Hdac3* depletion on skeletal maturation were determined by crossing *Hdac3^fl/−^* mice [11], [31] with mice expressing Cre recombinase from the *osterix (Osx)* promoter [30]. This breeding strategy eventually created five groups of progeny: wildtype (*Hdac3^fl/+^:Cre^−^*), heterozygotes (*Hdac3^+/−^: Cre^+ or −^ or Hdac3^ fl/−^:Cre^−^*; Hets), conditional heterozygotes (*Hdac3^fl/+^:Cre^+^*; CHet), conditional knockouts with a single germline null allele (*Hdac3^fl/−^:Cre^+^*; CKO), and osterix-driven CKO (*Hdac3 ^fl/fl^: Cre^+^*; CKO_Osx_) (Figure 1A). Immunoblot, qPCR, and microarray analyses of parietal bone protein and mRNA extracts from 4 to 5 day- old mice demonstrated that Hdac3 levels were significantly reduced in both groups of CKO mice (Figure 1B and Table 1). The expression of other Hdacs was not altered (data not shown). *Hdac3* CKO and CKO_Osx_ mice were indistinguishable in size at birth from wildtype and heterozygous littermates, but were smaller at the time of weaning and remained smaller throughout their lifespan, with male and female CKO animals being approximately 40% and 20% smaller than wildtype littermates, respectively (Figure 1C). Heterozygous mice were not significantly different in size and weight from wildtype animals (Figure S1). The *Hdac3* CKO and CKO_Osx_ mice had shorter lifespans with some mice dying as early as 3 weeks of age from undetermined causes (Figure 1D). The oldest CKO mouse survived 77 weeks, but was still outlived by heterozygous and wildtype mice.

**Table 1:** Genes Differentially Regulated in Calvarial Osteoblasts from Hdac3 CKO Mice.

Genes Upregulated in Hdac3 CKO Mice		Genes Downregulated in Hdac3 CKO Mice	
Gene Symbol	Fold Change	Gene Symbol	Fold Change
Cdkn1a	3.4 *	S100a8	−7.3 *
Ephx1	2.8 *	Alas2	−2.6 *
Phlda3	2.0 *	Ramp1	−2.3 *
Plat	2.0 *	Rsad2	−2.2 *
Tmem178	2.0 *	Mepe	−2.0 *
AB023957	1.9 *	Hdac3	−2.0 #
Gstm6	1.9 #	Serpina3g	−1.9 #
Nrp1	1.8 *	Vit	−1.9 #
Icam1	1.8 #	Hpgd	−1.8 #
Tmem43	1.7 #	CD59a	−1.7 #
Gpnmb	1.7 #	Entpd3	−1.6 #
LOC10004	1.7 #	Sct	−1.6 ∧
E430002G	1.7 #	Gpihbp1	−1.6 #
Slc19a2	1.6 #	Hey1	−1.6 #
Pax1	1.6 #	CD59b	−1.6 #
Gas6	1.6 #	Icam2	−1.6 #
Anxa8	1.6 #	Ankrd37	−1.5 #
Mmp2	1.6 #	Prr15	−1.5 ∧
Aaas	1.6 #	Cdk5r1	−1.5 #
Ahnak2	1.6 #	Ndufa1	−1.4 ∧
Timp3	1.6 #	Rbp7	−1.4 ∧
LOC10004	1.6 #		
Angptl4	1.6 #		
Slc16a9	1.6 #		
Slc29a3	1.6 #		
Tnfrsf11b/Opg	1.6 ∧		
Lgr5	1.6 #		
Scd2	1.5 #		
Prelp	1.5 #		
Bcl11b	1.5 #		
Cxcl2	1.5 #		
Exoc4	1.5 #		
Lgals3bp	1.5 #		
Prad1	1.5 ∧		
Tpm1	1.5 ∧		
Ror2	1.4 ∧		
Palld	1.4 #		
Sfrp4	1.4 ∧		
Col5a1	1.4 ∧		
Hmgcs2	1.4 ∧		
Polk	1.4 ∧		
Axin2	1.4 ∧		
Rnf145	1.4 ∧		

*3SD;

#>2 SD;

∧ >1 SD.

**Figure 2:**
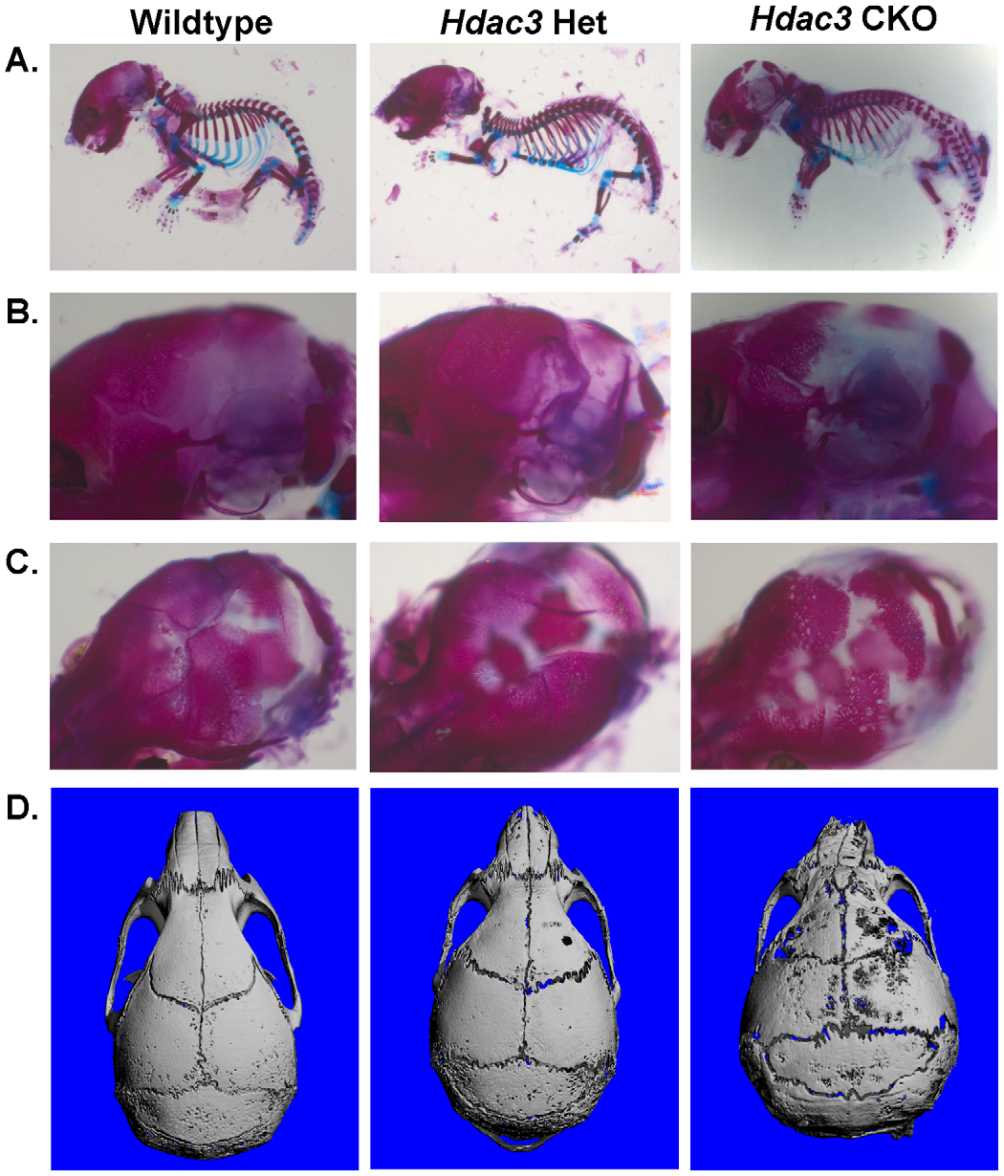
Calvarial bone formation is defective in Osx-Cre: *Hdac3* CKO mice. A–C. Staining of one day-old wildtype, het (*Hdac3^+/−^:Osx-Cre^−^*), and *Hdac3* CKO mice with alizarin red and alcian blue. D. MicroCT reconstructions of skulls from 5.5 week-old wildtype, Het (*Hdac3^+/−^: Osx-Cre^+^*), and *Hdac3* CKO mice.

The consequences of *Hdac3* depletion in osterix-expressing cells on skeletal development were examined by whole mount skeletal staining. One day-old *Hdac3* CKO mice exhibited grossly normal skeletal patterning with both cartilaginous and calcified skeletal elements as detected by Alician blue and Alizarin red dyes, respectively (Figure 2A). However, early calvarial development was impaired (Figure 2B–2D). Parietal bone formation was notably delayed in *Hdac3* CKO mice. Microcomputed tomography reconstruction of skulls from 5.5 week-old adult *Hdac3* CKO mice showed that the calvarial bones remained extremely thin and porous through adulthood (Figure 2D and Figure S2). These data indicated that Hdac3 is necessary for proper intramembranous bone formation.

### Hdac3 Conditional Knockout Mice Have Growth Plate Defects

**Figure 3:**
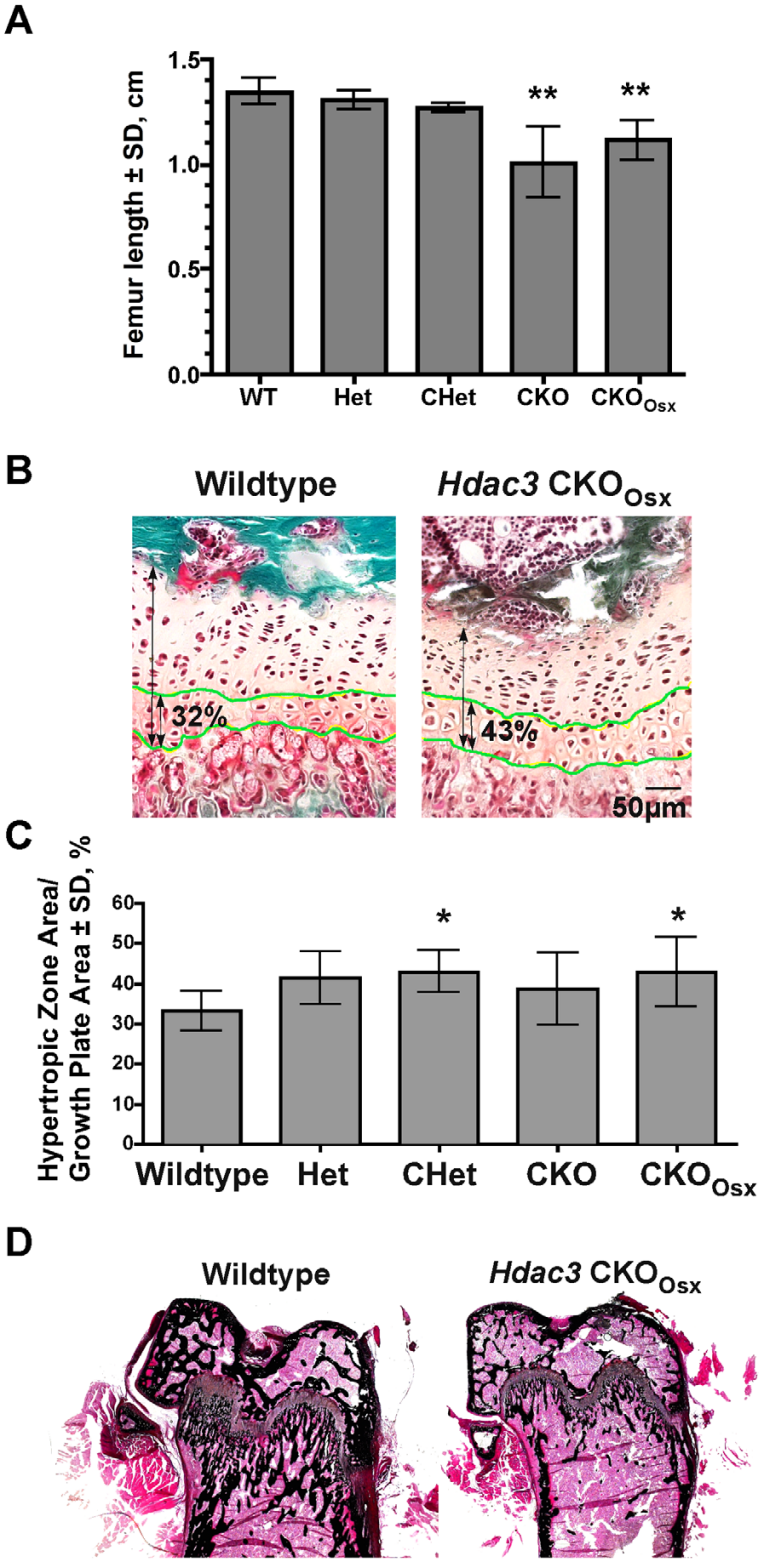
Growth plates are abnormal in Osx-Cre: *Hdac3* CKO mice. A. Femurs are shorter in male *Hdac3* CKO and CKO_Osx_ mice as compared to wildtype and heterozygous littermates at 5.5 weeks of age. (n = 11 WT, 3 Het, 5 CHet, 7 CKO, and 9 CKO_Osx_), **p≤0.01. B–C. The hypertrophic zone within growth plates of male mice was measured from Goldner's trichrome stained sections of the distal femurs. (n = 10 WT, 4 Het, 4 CHet, 6 CKO, and 6 CKO_Osx_), *p<0.05. D Von Kossa staining of representative distal femurs.

The smaller stature of the *Hdac3*-depleted mice and potential reduction of Hdac3 levels in cartilage due to osterix expression in chondrocytes [3], [32] prompted the assessment of femoral growth plate structure. Femur length was decreased 18 to 25% in male *Hdac3*-CKO and CKO_Osx_ mice (Figure 3A) and 17% in female mice (data not shown). The average growth plate depth of male 5.5 week-old *Hdac3*-CKO_Osx_ mice was the same size as the average from wildtype mice and had both proliferating and hypertrophic zones; however, the hypertrophic zone constituted a larger percentage of the growth plate in *Hdac3*- heterozygous and CKO groups than in wildtype mice (Figure 3B and 3C). Von Kossa staining revealed fewer trabeculi below the growth plate in 5.5 week-old CKO animals (Figure 3D). These results suggest that *Hdac3* deletion might delay or arrest terminal chondrocyte differentiation, which could adversely affect endochondral bone formation; however, the normal femur length of heterozygous animals despite an extended hypertrophic zone indicates a severe mineralization defect.

### Hdac3 Conditional Knockout Mice Have Low Trabecular Bone Density

**Figure 4:**
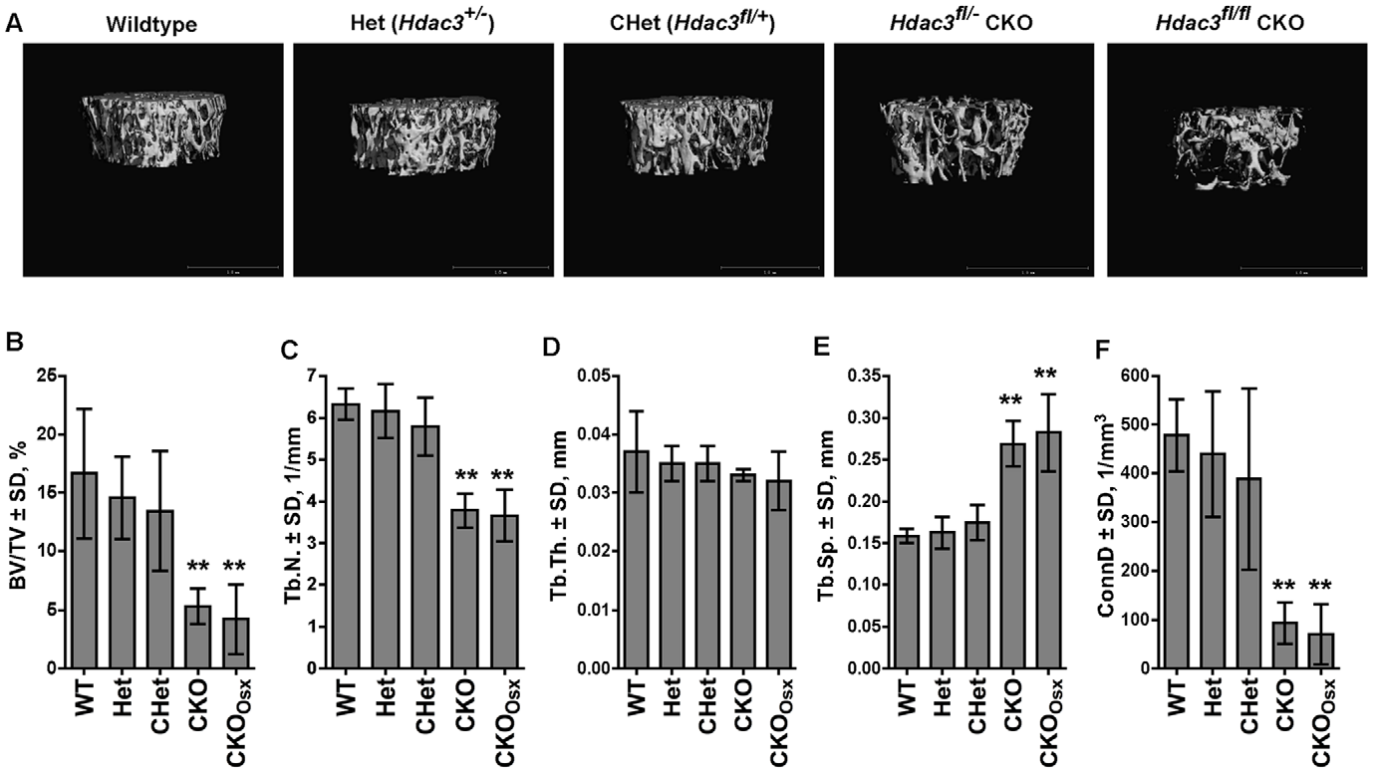
*Hdac3*-depletion in Osx-expressing cells decreases trabecular bone volume in distal femora. A. Representative microCT reconstructions of trabecular bone in distal femurs. B–F. Structural parameters of distal femurs from microCT reconstructions of 5.5 week-old male mice. n = 5 per group, **p<0.01.

The extent of trabecular bone loss in long bones from *Hdac3* CKO mice was measured by microcomputed tomography (Figure 4). Reconstructions of the trabecular bone in distal femurs illustrated the striking reduction in bone volume in both CKO and CKO_Osx_ mice as compared to their wildtype and heterozygous littermates (Figure 4A). At 5.5 weeks of age, trabecular bone volume density (BV/TV) in the distal femurs of male CKO mice was reduced by 75% (Figure 4B). The reductions were due to a 43% decrease in trabecular number (Tb.N) (Figure 4C). Trabecular thickness (Tb.Th) was unchanged (Figure 4D). In accordance with decreased trabecular number in both groups of *Hdac3* CKO mice, trabecular separation (Tb.Sp) was increased and connectivity density (ConnD) was decreased in these animals (Figures 4E–F). Similar differences were also observed in 12 week-old male mice (Figure S3) and in females (data not shown).

**Figure 5 pone-0011492-g005:**
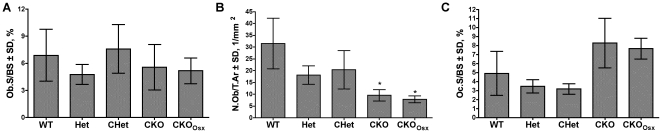
Osteoblast number is decreased in *Hdac3*-CKO mice. Distal femurs from 5.5 week-old male mice were sectioned and stained with Goldner's Trichrome (A and B) or TRAP (C). Osteoblast surface as a proportion of bone surface (Ob.S/BS; A), osteoblast number per tissue area (N.Ob/T.Ar; B) and osteoclast surface per bone surface (Oc.S/BS; C) were measured with Osteometrics software. n = 6−7, *p<0.05.

To understand how *Hdac3*-depletion in osterix-positive cells affected trabecular bone formation, Goldner's Trichrome-stained sections of distal femurs were examined by static histomorphometry (Figure 5). There was no change in the number of osteoblasts per bone surface (Figure 5A); however, in accordance with the significant decrease in bone density, there were fewer osteoblasts per tissue area in the *Hdac3* CKO mice as compared to wildtype and heterozygous littermates (Figure 5B). Osteoclast surface was slightly elevated in the *Hdac3* CKO mice; however the changes did not reach statistical significance (Figure 5C). These data indicate that *Hdac3* depletion in osterix-expressing cells is detrimental to bone formation, but that the bony structures present in these animals remained lined with osteoblasts and osteoclasts.

**Figure 6 pone-0011492-g006:**
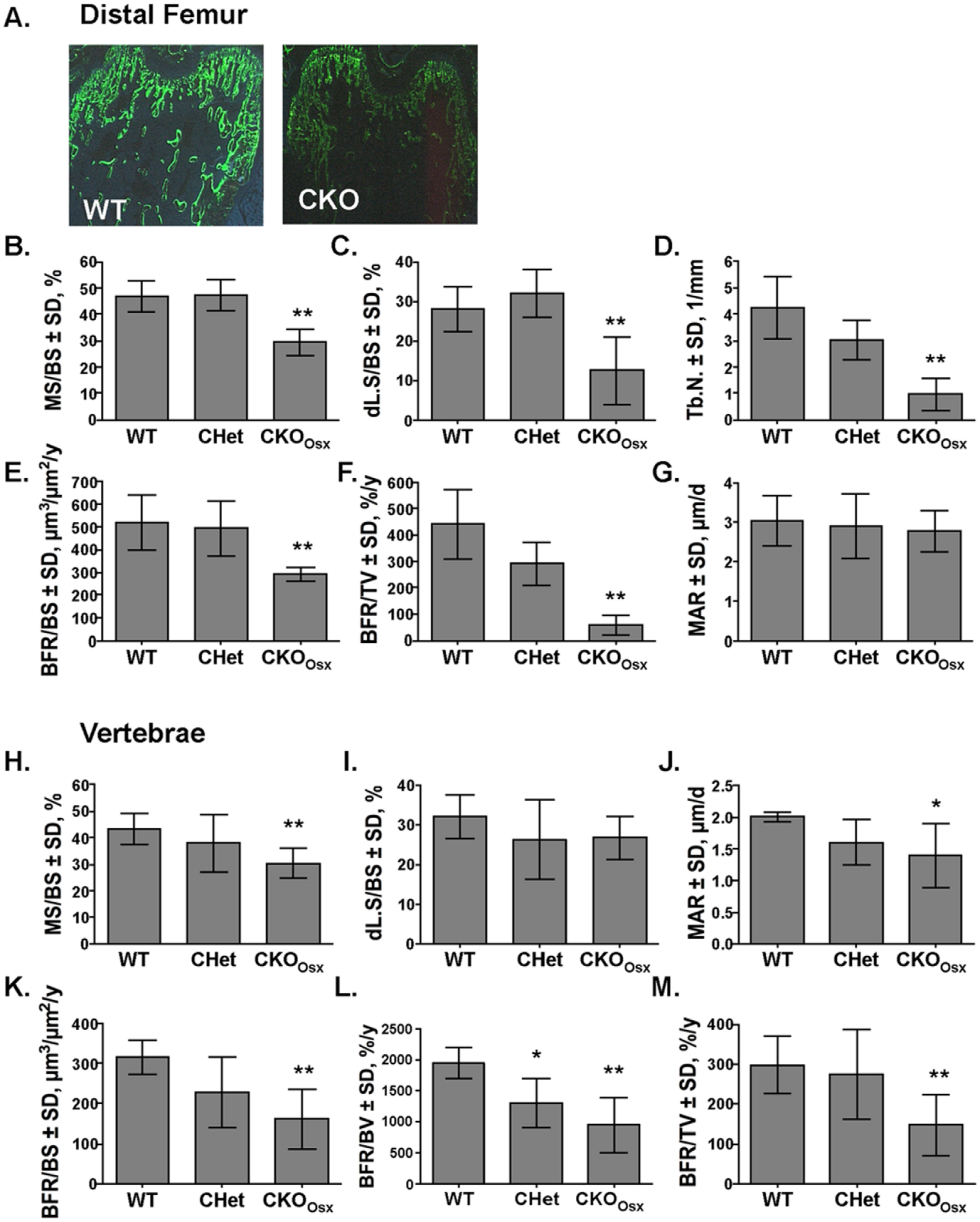
Trabecular bone formation rates are lower in *Hdac3* CKO mice. A Fluorochrome incorporation in the trabeculi of distal femurs is decreased in CKO mice. Newly formed trabecular bone in distal femurs (B–G) and vertebrae (H–M) was assessed in 5.5 week old male mice following two calcein injections using Osteometrics™ software. (n = 5), *p<0.05, **p≤0.01.

To determine if osteoblasts in *Hdac3* CKO_Osx_ mice produce a mineralized matrix at the same rate and to the same extent as wildtype and heterozygous littermates, 5.5 week-old male mice were injected once with tetracycline and twice with calcein and newly formed mineralized surfaces were measured (Figure 6A). The amount of mineralized and double-labeled surface (MS and dL.S, respectively) per bone surface (BS) were reduced by 38% and 55%, respectively, in the distal femurs of *Hdac3* CKO_Osx_ mice (Figures 6B and C). Similar to what was observed in the microCT scans (Figure 4D), trabecular number was decreased in histological sections (Figure 6D). Trabecular bone formation rates (BFR) per bone surface and tissue volume were also significantly reduced by 44% and 87%, respectively, in *Hdac3* CKO_Osx_ mice (Figures 6E and F). However, mineral apposition rate (MAR), which is defined as the distance between two labels formed over a defined period of time, was not altered by *Hdac3* deficiency in distal femurs (Figure 6G). Thus, in the rare places where bone was present and two labels were detected on a bone surface, the activity of osteoblasts in the *Hdac3* CKO_Osx_ mice was indistinguishable from that of wildtype mice. Reductions in mineralized surface (30%) and bone formation rates (50%) were also observed in vertebral trabeculi (Figures 6H–L). The MAR in vertebrae was reduced by 30% (Figures 6J). Similar results were present in the CKO (*Hdac3^fl/−^:Cre^+^*) mice (data not shown). Together, these data indicate that there are widespread deficits in trabecular bone formation in *Hdac3* CKO mice.

### Hdac3 Conditional Knockout Mice Have Low Cortical Bone Density

**Figure 7:**
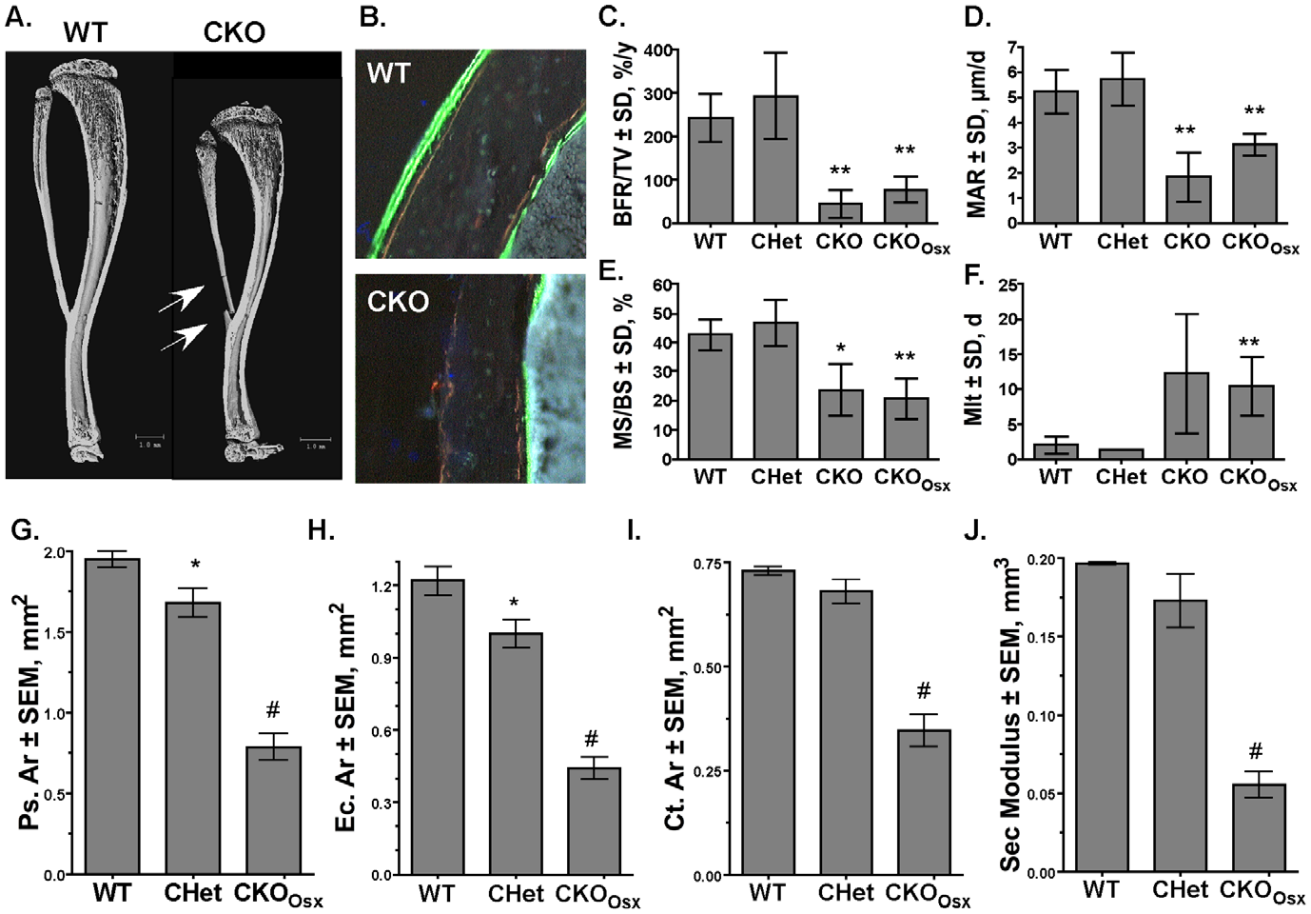
Cortical bone is thinner in *Hdac3* CKO mice. A. MicroCT reconstructions of tibia and fibulas from WT and *Hdac3* CKO mice. Arrows point to fractures. B–F. Calcein incorporation (B), bone formation rates (C), mineral apposition rates (D), mineralized surfaces (E) were decreased, while mineralization lag time (F) was increased in *Hdac3* CKO mice (n = 5 WT and CKO_Osx_, n = 2 CHet, n = 3 CKO). G–J. Cortical measurements from microCT reconstructions demonstrate that periosteal area (Ps.Ar, G), endocortical area (Ec.Ar, H), Cortical area (Ct.Ar), which estimates axial strength (I), and section (Sec) modulus, an estimate of bending strength (J), were reduced in *Hdac3* CKO mice (n = 3 per group). *p<0.05, **p<0.01, #p<0.0001.

Few naturally occurring fractures were observed in *Hdac3* CKO and CKO_Osx_ mice; however, the long bones broke frequently along the diaphysis during dissection and processing (Figure 7A), suggesting cortical bone defects. Fluorochrome-labeling experiments demonstrated dramatic defects in bone formation on the periosteal and endocortical bone surfaces in both groups of *Hdac3* CKO mice (Figure 7B). The bone formation rate, mineral apposition rate, and mineralized surface percentage were all significantly decreased in both CKO and CKO_Osx_ mice (Figure 7C, 7D, and 7E). Meanwhile, mineralization lag time was increased 5- to 6-fold in the CKO groups (Figure 7F). Periosteal and endocortical areas at the mid-diaphysis of the femur were reduced more than 60% in the *Hdac3* CKO_Osx_ mice (Figures 7G and 7H). Cortical area and section modulus, which are estimates of axial strength and bending strength [33], were reduced 50 and 70%, respectively, in the *Hdac3* CKO_Osx_ mice (Figures 7I and 7J). Together the data presented thus far demonstrate that *Hdac3*-deficiency prevents proper calvarial, trabecular, and cortical bone formation.

### Adipogenesis is Increased in Bone Marrow of Hdac3 Conditional Knockout Mice

**Figure 8:**
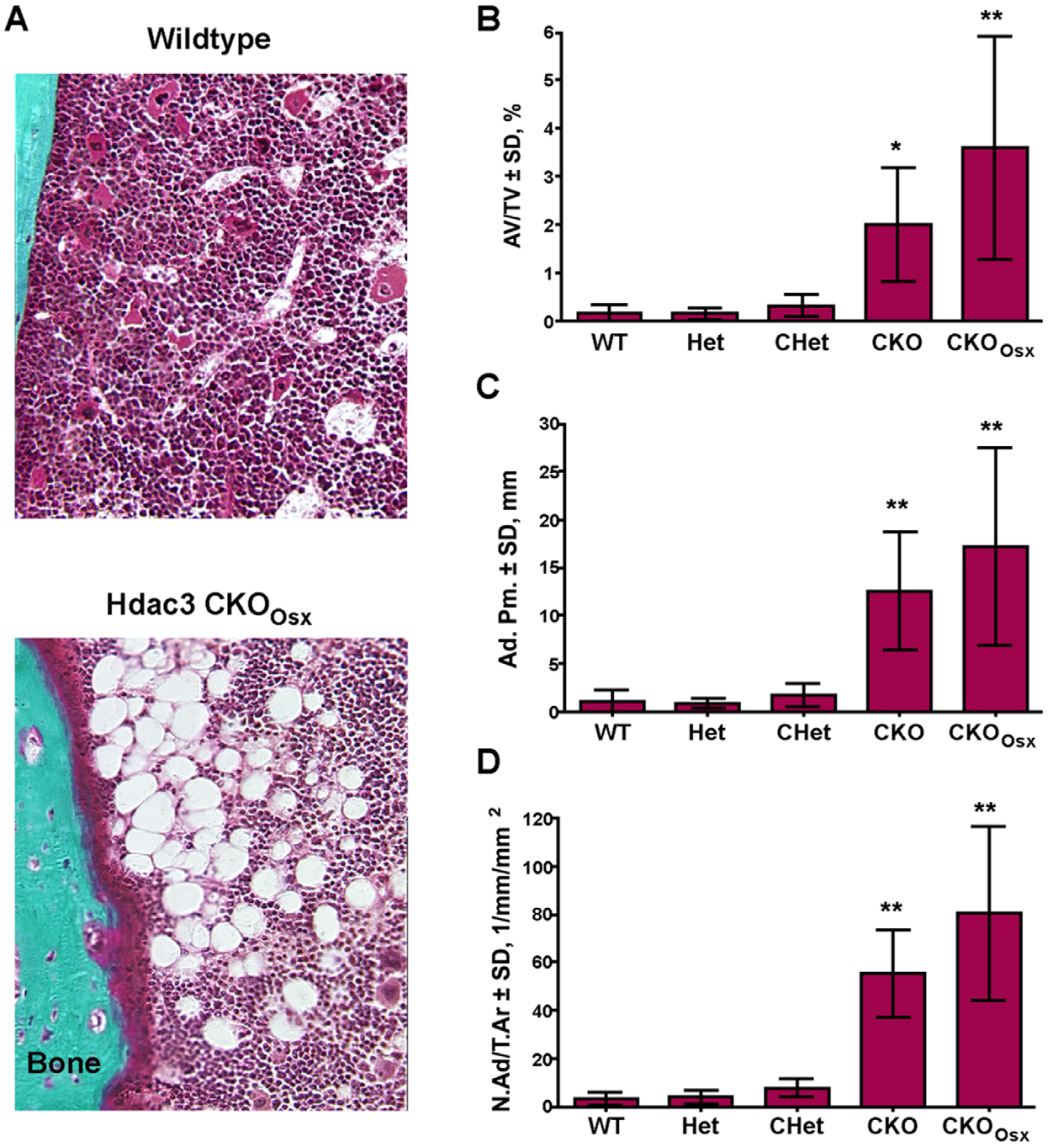
*Hdac3*-depletion in osterix-expressing cells increases adipocyte volume within bone marrow. A. Goldner's Trichrome stain of femurs from 5.5 week-old male wildtype and *Hdac3*-CKO mice. B–D. Adipocyte density (Adipocyte volume/tissue volume (B), perimeter (C), and number (D) are increased in *Hdac3* CKO mice. n = 4−6, *p<0.05, **p<0.01.

Bone marrow osteoblasts and adipocytes are derived from common multipotent mesenchymal progenitor cells and an inverse correlation between bone and white fat volumes are well characterized [34]. Goldner's trichrome-stained sections from 5.5 week-old *Hdac3* CKO femurs revealed the presence of a large number of adipocyte ghosts (Figure 8A). The adipocytes were present throughout the femur but seemed to be more prevalent towards the midshaft and along cortical surfaces. Adipocyte density, perimeter and number were elevated 11- to 25-fold in both CKO and CKO_Osx_ groups, but not in Het or CHet mice (Figure 8B–D). Similar increases in adipocyte density were observed in female animals and in 2 and 4 week-old mice (data not shown). Adipocytes were not seen in 4 day-old mice (data not shown), indicating that bone marrow adipogenesis resulting from *Hdac3*-deficiency occurred postnatally.

### Microarray analysis of Osteoblasts from Hdac3 CKO mice

**Figure 9:**
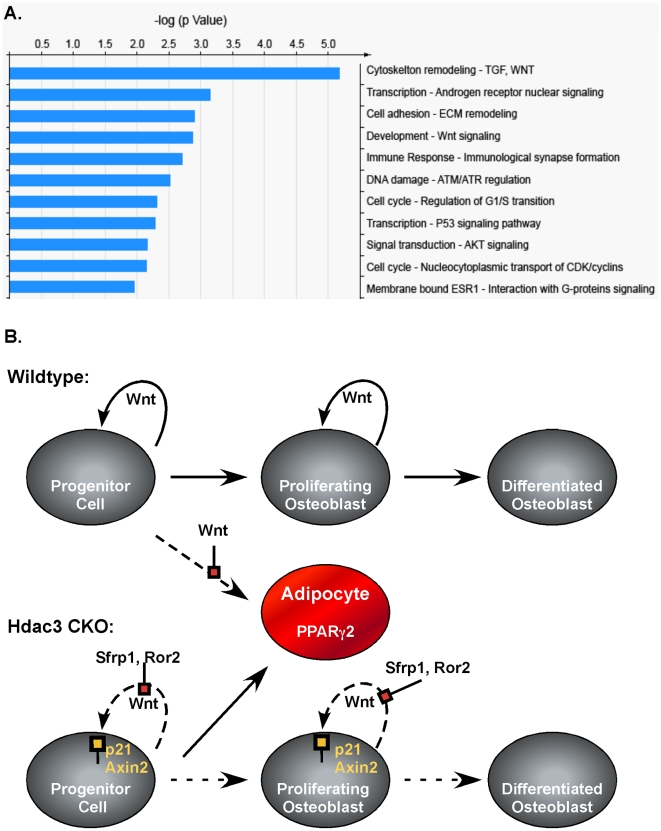
*Hdac3* depletion alters multiple signaling pathways. A. GeneGo pathway analysis of genes differentially regulated in *Hdac3*-deficient calvarial osteoblasts. Data from two independent microarray experiments were analyzed with MetaCore software at a false discovery rate = 0.25. B. Model for defective osteoblast differentiation and increased adipocyte formation in *Hdac3*-insufficient mice.

To understand the molecular changes that result from *Hdac3* depletion in osteoblast progenitor cells, the gene expression patterns in calvarial extracts were determined by microarray analysis and confirmed by qPCR. Two independent microarray analyses were performed with eight paired wildtype and CKO or CKO_Osx_ littermates. In these experiments, 768 and 786 genes were significantly changed (fold change >1.1, p<0.05 and 95% confidence) in the *Hdac3* CKO_Osx_ mice, but only 176 genes were present in both data sets (Table S1). Expression changes of 1.4 fold or greater were verified by qPCRs. With this more stringent cutoff (fold change >1.4), 64 genes were identified (Table 1). Of these, 43 genes were upregulated in *Hdac3* CKO mice and 21 genes were downregulated. As expected, *Hdac3* was one of these most suppressed genes. The most upregulated gene in both experiments was cyclin-dependent kinase inhibitor 1a (Cdkn1a, also known as p21^Cip1^), a known Hdac regulated gene [35], [36], [37]. In reporter assays we found that Hdac3 repressed the p21(WAF1/CIP1) promoter in a concentration dependent manner (Figure S4). Gene ontogeny analysis of genes differentially regulated more than 1.4-fold identified 11 pathways as being affected by *Hdac3* deletion (Figure 9). These pathways include signal transduction cascades (e.g. Wnt, Akt, G-proteins), cell cycle and DNA damage pathways, and cytoskeletal/cell adhesion. Notably, osterix mRNA levels were unchanged (data not shown). Thus, *Hdac3* depletion modulates several pathways that are crucial for osteoblast development and function.

## Discussion

Hdac3 is essential for embryonic development [11], [12]. It associates with numerous transcription factors, co-repressors and class II Hdacs [18], [19], [20], [21], [22]. We previously showed that Hdac3 binds and represses Runx2-dependent activation of osteoblast genes [24]. In this study, we demonstrate that conditional deletion of *Hdac3* in cells where Cre recombinase is expressed from the endogenous osterix promoter causes severe deficits in calvarial, trabecular and cortical bone volume. Some *Osx-Cre* expressing mice (*Hdac3* wildtype) also had mild skull defects (Figure S5); however, all *Hdac3* CKO mice had severe craniofacial abnormalities. Importantly, none of the most differentially regulated genes (Table 1) were significantly changed in *Osx-Cre* mice with wildtype *Hdac3* alleles. Interestingly, incomplete skull bone ossification was also observed in fetal rats exposed to the Hdac inhibitor vorinostat *in utero* [38]. Distal femur trabecular bone volumes were not affected by the expression of Cre or loss of a single *Hdac3* allele (e.g. CHet mice), but were severely reduced when both copies of *Hdac3* were inactivated. Trabecular and cortical bone formation rates were markedly depressed in both groups of CKO mice (*Hdac3^fl/fl^* ^or^ *^fl/−^*) with concomitant increases in bone marrow fat. Together these data demonstrate that Hdac3 is essential for proper osteoblast function and bone formation.

In this study we focused on the postnatal consequences of *Hdac3* depletion on bone structural and cellular properties. Because the *Hdac3* CKO mice were smaller than wildtype and heterozyogous littermates, we also examined growth plates in skeletally immature mice. The hypertrophic zone was thicker in *Hdac3*-haploinsufficient and conditional knockout animals; suggesting that Hdac3 activity might be necessary to complete chondrocyte differentiation, promote apoptosis, and/or for the onset of ossification. Interestingly, only animals in the CKO groups (not the Het or CHet groups) exhibited significant changes in trabecular and cortical bone structure (Figures 4–7), which likely explains their smaller stature. Preliminary studies suggest that endochondral ossification of the bone collar was delayed in neonatal *Hdac3* CKO mice (data not shown). More focused studies are in progress to determine how Hdac3 participates in the temporal events of endochondral bone formation.

Two p53 inducible genes, p21^CIP1^ and Phlda3 [39], [40], were upregulated in *Hdac3* CKO mice. This raises the possibility that DNA damage might be contributing to defective osteoblast maturation in the CKO animals (Figure 9B). The cyclin-dependent kinase inhibitor, p21 (CIP1/WAF1), was the most upregulated gene in *Hdac3* CKO mice and Hdac3 repressed the basal activity of the p21^Cip1^ promoter fragment. We previously showed that Runx2 represses p21^Cip1^ via Hdac-dependent mechanisms in osteoblasts [25]. p21^Cip1^ was one of the first genes described to be upregulated by Hdac inhibitors [35], [36], [37] and is elevated in *Hdac1*-deficient embryonic stem cells, *Hdac2* KO mice [9], [41] and in liver-specific *Hdac3* CKO mice [31]. Overexpression of p21 in osteoblasts blocked proliferation and differentiation [42]. Thus, elevated levels of p21 may interfere with osteoblast maturation and activity in our *Hdac3* CKO mice.

Several other genes and signaling pathways that were affected by *Hdac3* depletion have known roles in osteoblast maturation. Notably, antagonists of matrix mineralization, matrix gla protein (Mgp) and ectonucleotide pyrophosphatase (Enpp)1[43], [44]were elevated. We did not observe changes in osteocalcin levels; however, osteoblast lineage cells are indeed inhibited as a result of *Hdac3* depletion as evidenced by reduced bone formation rates and the downregulation of genes (e.g. MEPE) expressed by mature osteoblasts. Inhibitors (Axin2, Ror2, Sfrp4) of the canonical Wnt signaling pathways were also increased (Figure 9B). Suppression of Wnt10b signaling may decrease the osteogenic potential and increase adipogenesis of multipotent mesenchymal cells [45]. Additional experiments beyond the scope of this study are needed to determine how canonical Wnt signaling is affected by *Hdac3*-depletion.

Conditional *Hdac3* deletion disrupts metabolic pathways and activates Ppar nuclear receptors. For example, elimination of *Hdac3* in postnatal liver cells increased the expression of Ppar-gamma 2 target genes and caused lipid accumulation, hepatocyte hypertrophy and hepatomegaly [31]. Conditional deletion of *Hdac3* in cardiomyocytes elevated expression of Ppar-alpha target genes and increased fatty-acid-induced myocardial lipid accumulation and cardiac hypertrophy [12]. Another mouse model, which globally disrupted the interaction between Hdac3 and Ncor1 (an activating component of Hdac3 protein complexes), revealed that this protein pair contributes to circadian and metabolic physiology [46]. The data reported within show that *Hdac3*-depletion in osterix-positive progenitor cells impairs the activities of osteoblasts derived from these multipotent cells and promotes adipocyte differentiation. Osterix was previously described as a product of preosteoblasts and immature chondrocytes [3], [32]. Our data suggest that some of these cells retain the potential to differentiate into the adipocyte lineage in vivo. The absence of *Hdac3* may promote the activity of Ppar-gamma 2, which is an essential transcription factor for adipogenesis and binds to Hdac3-containing co-repressor complexes [47]. Several Ppar-gamma 2-regulated genes (e,g., Angiopoietin-like 4, Mmp2 [48], [49]) were increased in CKO mice (Table 1). Elevated serum levels of adiponectin, another Ppar-gamma 2-activated gene, in *Hdac3* CKO mice are also consistent with increased Ppar-gamma2 activity (data not shown). Together, the existing mouse models agree that Hdac3 is a crucial regulator of lipid accumulation in multiple tissues.

Multiple Hdacs, including Hdac3, regulate osteoblast maturation in vitro [24], [26], [27], [29], [50]. RNAi-mediated Hdac3 suppression in an immortalized and lineage-committed calvarial cell line, MC3T3-E1, promoted calcium deposition in the extracellular matrix, and increased the expression of osteoblast maturation genes [24]. Broad-acting Hdac inhibitors also promoted osteogenic maturation of several cell lines, primary calvarial cells and bone marrow-derived mesenchymal progenitor cells [28], [50], [51], [52], [53]. Thus, the results of this study are inconsistent with several *in vitro* studies. An explanation for this discrepancy is that Cre-mediated recombination of *Hdac3* in progenitor cells is a permanent event. By comparison, small molecule Hdac inhibitors have short half-lives in serum and might only temporally change chromatin structure and gene expression in relatively short-term cell culture assays. Moreover, non-proliferating cells and cells with intact cell cycle checkpoints are resistant to the toxic effects of Hdac inhibitors [11], [54]. However, this population of cells may still be susceptible to the sustained effects of *Hdac3* deletion in vivo. Thus, the fact that the decreased osteoblast viability we observed with the in vivo knockout of Hdac3 was not seen after siRNA suppression of Hdac3 in vitro may be due to differences in the composition and/or maturity of the targeted cell populations as well as to the methods used to reduce Hdac3 expression or function.

In conclusion, Hdac3 is essential for bone formation. Conditional deletion of *Hdac3* with osterix-driven Cre recombinase drastically reduces trabecular and cortical bone formation rates, bone volume and trabecular number while increasing bone marrow adipogenesis. Preliminary experiments show that conditional *Hdac3* deletion with other osteoblast-specific Cre drivers (i.e. Col1a1 and osteocalcin) also causes bone loss. These results are consistent with our recent studies examining how the Hdac inhibitor vorinostat (SAHA) affects skeletal health in a preclinical cancer model. Although vorinostat inhibited tumor burden in murine long bones, bone mass of contralateral limbs was significantly reduced [55]. Vorinostat represses multiple Hdacs and the data reported here suggest that *Hdac3* suppression is sufficient to decrease bone volume. Our results indicate that sustained Hdac3 suppression is likely to produce osteopenia and potentially fractures. Hdac inhibitors are in many clinical cancer trials and vorinostat is already approved in the United States for treating cutaneous T cell lymphomas [56]. Hdac inhibitors are also being considered as possible therapies for several non-cancerous conditions, including degenerative and inflammatory disorders [57]. Our data strongly suggest that bone density should be monitored in any individuals receiving Hdac inhibitors as therapy. Moreover, future generations of Hdac inhibitors should be designed to avoid targeting Hdac3 because of multiple organ (heart, liver and bone) failures in *Hdac3*-deficient mice [12], [31].

## Materials and Methods

### Mice/Genotyping

Mice were genotyped for *Hdac3* alleles as reported [11]. Osx-Cre transgenic animals were previously described [30]. Animals were housed in an accredited facility under a 12-hour light/dark cycle and provided water and food (PicoLab® Rodent Diet 20, LabDiet) ad libitum. Diets were supplemented three times per week with Nutri-Cal® (Vétoquinol Canada Inc, Lavaltrie, QC) and Napa Nector (SE Lab Group Inc, Napa, CA). For dynamic histomorphometry experiments, 5.5 and 12 week-old mice were injected subcutaneously with tetracycline (25 mg/kg) 7 and 14 days, respectively, before euthanasia. They were also injected subcutaneously twice with calcein (10 mg/kg) at four days and one day before ending the experiment. All animal research was conducted according to guidelines provided by the National Institute of Health and the Institute of Laboratory Animal Resources, National Research Council. The Mayo Clinic Institutional Animal Care and Use Committee approved all animal studies.

### Whole Mount Embryo Staining

Newborn pups were euthanized and placed in chloroform. Skeletons were dissected and fixed overnight in ethanol. Cartilage elements were stained with a 30% Alcian blue dye (dissolved in 80 ml 95% ethanol and 20 ml glacial acetic acid). Skeletons were washed twice with 95% ethanol and then placed in 2% KOH until the remaining soft tissues were dissolved. Bones were stained with 75 µg/ml Alizarin red S (Sigma) in 1% KOH overnight and then destained in 20% glycerol, 1% KOH for 2 weeks, with daily solution changes. Skeletons were transferred to a 20% glycerol, 20% ethanol solution overnight and then stored in a 50% glycerol, 50% ethanol solution indefinitely.

### Microcomputed Tomography

Bones were dissected from euthanized mice and fixed in 70% ethanol. Samples were scanned and measured in a Scanco 35 System (Scanco Medical, Switzerland), which was calibrated monthly with an aluminum rod phantom and weekly with a hydroxyapatite phantom. Images were reconstructed with cubic voxels 5.7 µm on a side using a modified Feldkamp cone beam tomographic reconstruction algorithm. Trabecular bone properties were calculated with the manufacturer's software. Cortical bone geometric properties were measured from femoral mid-shaft cross-sectional images as previously described [58]. Data from *Hdac3* mutant mice were compared to data from wildtype mice with student t-tests.

### Histomorphometry

After microcomputed tomography scanning, two transverse sections (∼50 microns thick) were cut from the diaphysis of long bones using an Isomet saw with a diamond wafer blade for cortical studies. The sections were polished and ground to a thickness of approximately five microns with a glass plate coated in aluminum oxide powder, mounted on a microscope slide, and examined with a fluorescent microscope. For trabecular studies, distal femurs were dehydrated, embedded in methylmethacrylate and cut longitudinally into five micron-thick sections. The dynamic properties of trabecular bone surfaces were assessed on unstained sections at 200X using the OsteoMetrics™ system by measuring the distance between the two calcein labels, while the properties of the cortical periosteal and endosteal surfaces were determined by measuring the distance between the tetracycline label and second calcein label as previously described [59].

For static measures, the distal femurs were embedded in methylmethacrylate and cut longitudinally into five micron-thick serial sections. Serial sections were stained with Goldner's trichrome or von Kossa to detect mineralized bone, osteoid, cells and adipocytes; toluidine blue to detect osteoblasts; or TRAP (to distinguish osteoclasts) for static histomorphometric measures. Data were collected at 200× magnification using OsteoMeasure software. Data from *Hdac3* mutant mice were compared to those from wildtype mice with student t-tests.

### Growth Plate Analyses

Femur lengths were measured from the inter-condylar notch to trochanteric notch using a caliper. The distal femurs of 12-5.5 week-old male mice were embedded in methylmethacrylate and cut longitudinally into five micron-thick serial sections. Serial sections were stained with Goldner's trichrome. A technician blind to sample identities measured growth plate depth (µm) and hypertrophic zone depth (µm) with a previously published technique that was adapted for the KS300 computerized image analysis system (Carl Zeiss Vision GmbH, Hallbergmoos, Germany) [60], [61]. Briefly, two sections were obtained from each tissue sample and scanned at the growth plate area using the Zeiss AuxioCam MRc at the same magnification with the calcification zone horizontal to the bottom edge of the scan's frame of reference. Each growth plate or hypertrophic zone was outlined by hand. Average thickness was determined by comparing thicknesses in the outlined area from top to bottom. Values obtained from sections made from the same tissue sample were then averaged to give overall mean values for cambium thickness and hypertrophic zone thickness. Hypertrophic zone thickness was then normalized to growth plate thickness in order to yield percentage hypertrophic zone in growth plate. Statistical analysis was done using a student's t test and Wilcoxon Rank Sum non-parametric where appropriate. Statistics are reported with standard deviations.

### Calvarial Explants

To enrich for cellular protein and obtain RNA from bone tissue, calvarial bones from four to five day-old pups were dissected from soft tissues, rinsed with HBSS, and digested in collagenase digestion medium [52] on an orbital shaker for 20 minutes at 37 degrees at 125 rpm. Following digestion, explants were washed once with PBS and snap frozen in liquid nitrogen. The remaining bone tissues were crushed under liquid nitrogen with a mortar and pestle and then resuspended in either TRIzol (Invitrogen) for RNA extraction or modified-RIPA buffer supplemented with protease inhibitors for protein isolation.

### Microarray studies

RNA from calvarial explants was purified using TRIzol according to the manufacturer's protocol (Invitrogen) and reverse transcribed using Qiagen's Quantitect Reverse Transcription Kit. Microarray experiments were performed in the Mayo Clinic Advanced Genomic Technology Center using the Illumina MouseRef-8 BeadChip array. Two independent experiments were performed; each experiment used RNA from four wildtype and four CKO litter-matched mouse calvaria. The raw data from eight samples in each experiment were pre-processed using Beadstudio (Illumina Inc, San Diego, CA). Raw data were submitted to the MIAME-compliant GEO database (Accession number: GSE21087). Genes that were not expressed in eight calvarial RNA samples (detection p≥0.05) were eliminated from analysis, leaving 10,500 gene transcripts out of 19,000 for the final analysis. A paired t-test was used to compare differential gene expression between the wildtype and CKO groups. Genes with fold changes greater than 1 standard deviation in both experiments were used for pathway analysis using MetaCore software (GeneGo Inc, St. Joseph, MI, USA). Genes that were significantly (1.4 fold, p≤0.05) differentially expressed between the WT and CKO groups were subjected to reverse transcription (RT) and real-time semi-quantitative (q) PCR analysis for confirmation.

### RT-qPCR

To verify microarray results, calvarial RNA samples from litter-matched wildtype and CKO mice were reverse-transcribed with Quantitect Reverse Transcription Kit (Qiagen). Gene specific primers sequences are available upon request. Real time qPCRs were performed using 15 ng of cDNA per 10 µl using Bio-Rad iQ SYBR Green Supermix in the Eppendorf epMotion 5070 and the Applied Biosystems 7900 HT Fast Real Time PCR System. Transcript levels were normalized to three housekeeping genes (YWHAZ, GAPDH, and HRPT1), which were our most stable osteoblast genes as determined using GeNorm software [62]. Quantification was done using the 2^−ΔΔCt^ method [63].

### Western blotting

Protein extracts from calvarial explants were sonicated and resolved by SDS-PAGE. Immunoblotting was performed with antibodies recognizing Hdac3 (1∶1000, Abcam, Ab63353).

### Luciferase Assays

p21 reporter assays were performed as previously described [25].

## Supporting Information

Figure S1 Photograph of representative 5.5. week-old mice used in this study. The animals' weights are listed below the photos.
(0.57 MB TIF)

Figure S2 MicroCT Reconstructions of skulls from 5.5 week-old male Hdac3 WT, Het and CKO mice. These are alternative angles of the microCT reconstructions shown in Figure 1E.
(0.96 MB TIF)

Figure S3 Hdac3-Depletion in Osx-expressing cells decreases trabecular bone volume in distal femora. A-E. Structural parameters of distal femurs from microCT reconstruction of 12 week-old male mice. n = 4-5 per group, *p<0.05.
(0.10 MB TIF)

Figure S4 HDAC3 represses the p21(CIP1) promoter. C2C12 cells were transfected with a p21-luciferase reporter and increasing concentrations of pCMV-HDAC3. Firefly luciferase activity was normalized to renilla-luciferase activity to control for transfection efficiency. Data are shown relative to cells transfected with an empty CMV vector.
(0.04 MB TIF)

Figure S5 MicroCT Reconstructions of skulls from Osx-Cre mice. Minor craniofacial defects are present in 5.5 week-old Osx-Cre mice. These mice are wildtype at the Hdac3 loci.
(0.35 MB TIF)

Table S1 List of the 176 genes common to two independent microarray experiments.
(0.14 MB PDF)
