# Supplementary figures and images for: Histone Deacetylase 3 Depletion in Osteo/Chondroprogenitor Cells Decreases Bone Density and Increases Marrow Fat
Source: PLoS One. 2010 Jul 9;5(7):e11492. doi: 10.1371/journal.pone.0011492 (PMC2901996; doi:10.1371/journal.pone.0011492)

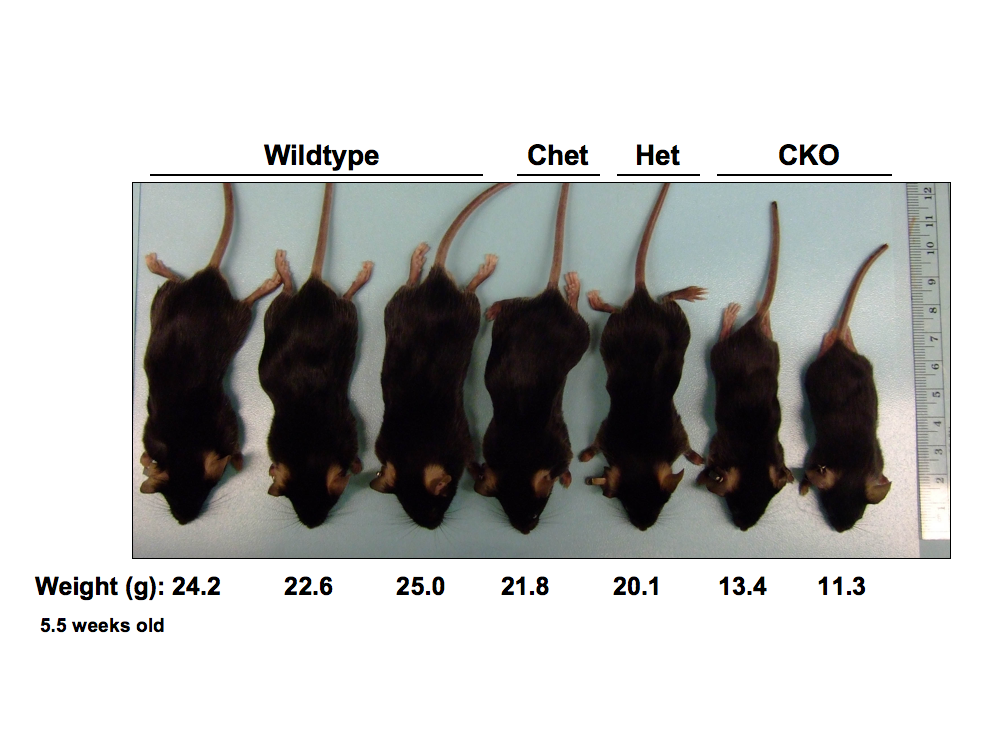

Supplement: Figure S1 — Photograph of representative 5.5. week-old mice used in this study. The animals' weights are listed below the photos. (0.57 MB TIF) [file pone.0011492.s001.tif]

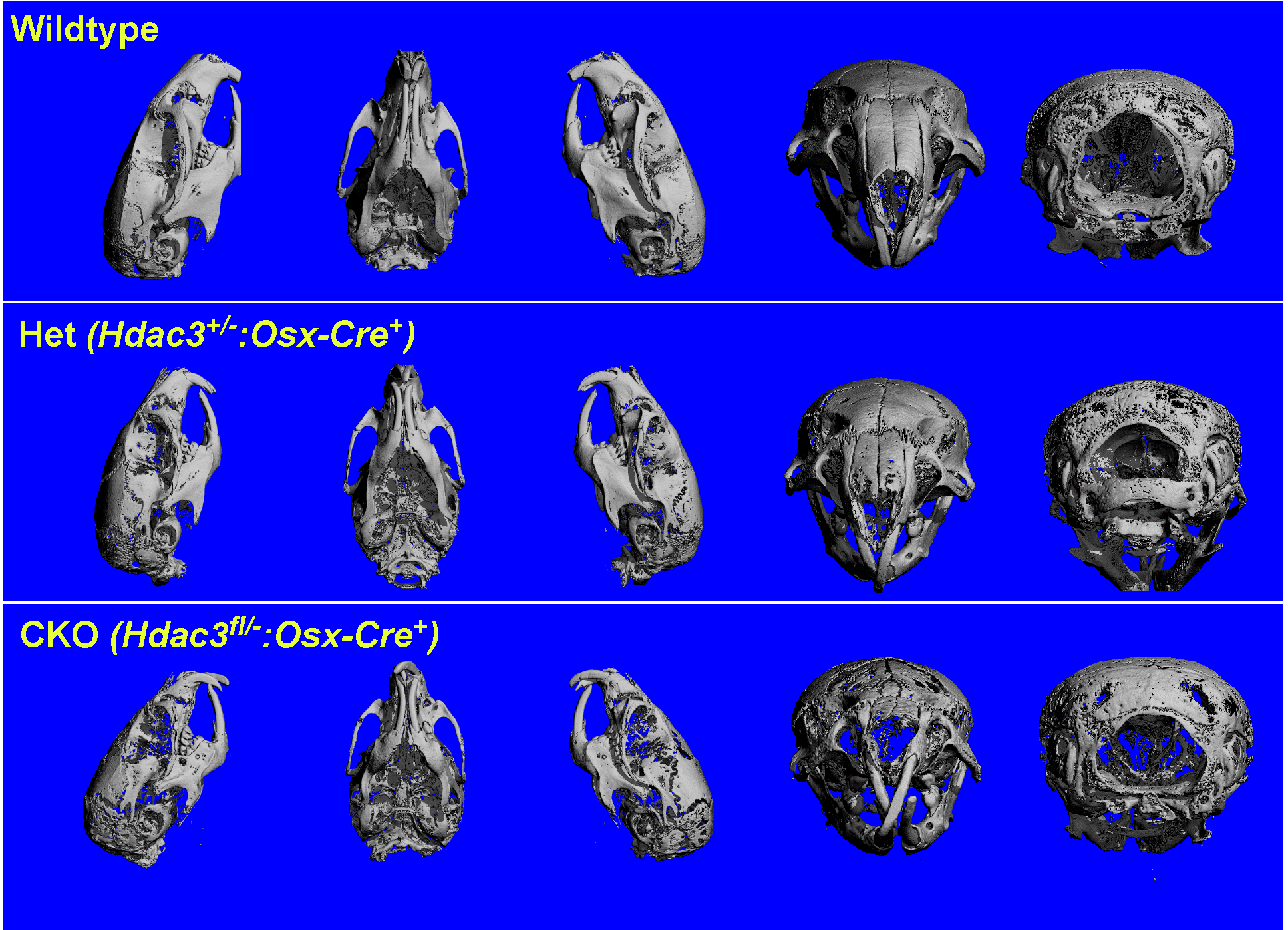

Supplement: Figure S2 — MicroCT Reconstructions of skulls from 5.5 week-old male Hdac3 WT, Het and CKO mice. These are alternative angles of the microCT reconstructions shown in Figure 1E. (0.96 MB TIF) [file pone.0011492.s002.tif]

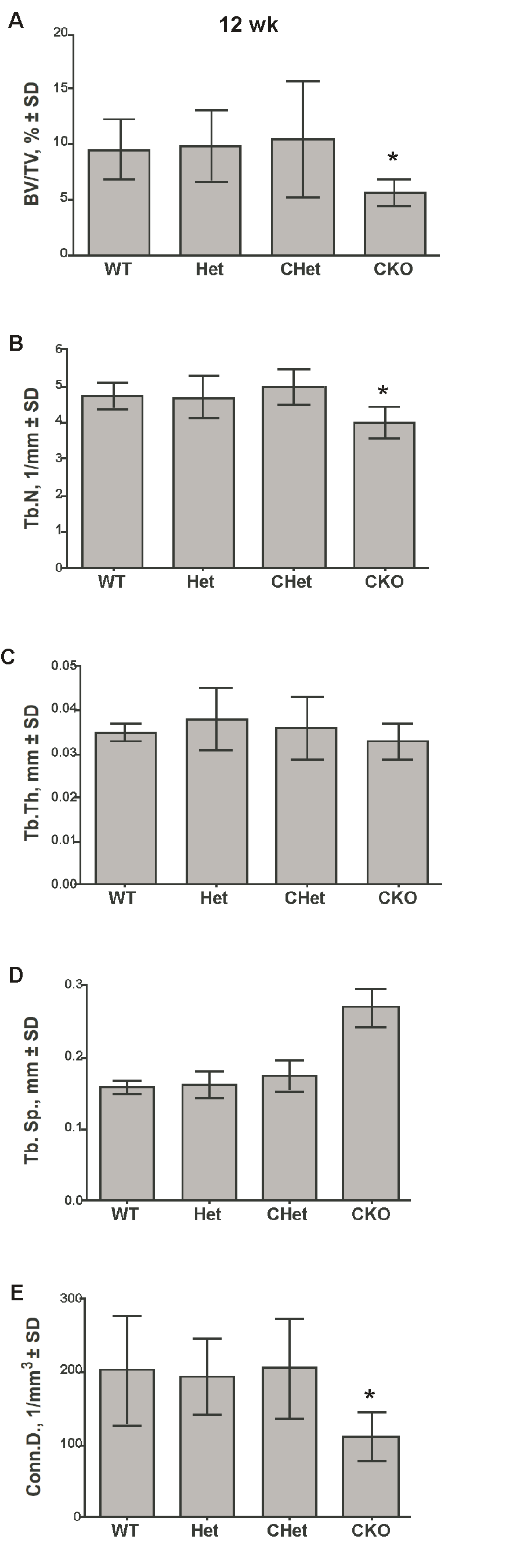

Supplement: Figure S3 — Hdac3-Depletion in Osx-expressing cells decreases trabecular bone volume in distal femora. A-E. Structural parameters of distal femurs from microCT reconstruction of 12 week-old male mice. n = 4-5 per group, *p<0.05. (0.10 MB TIF) [file pone.0011492.s003.tif]

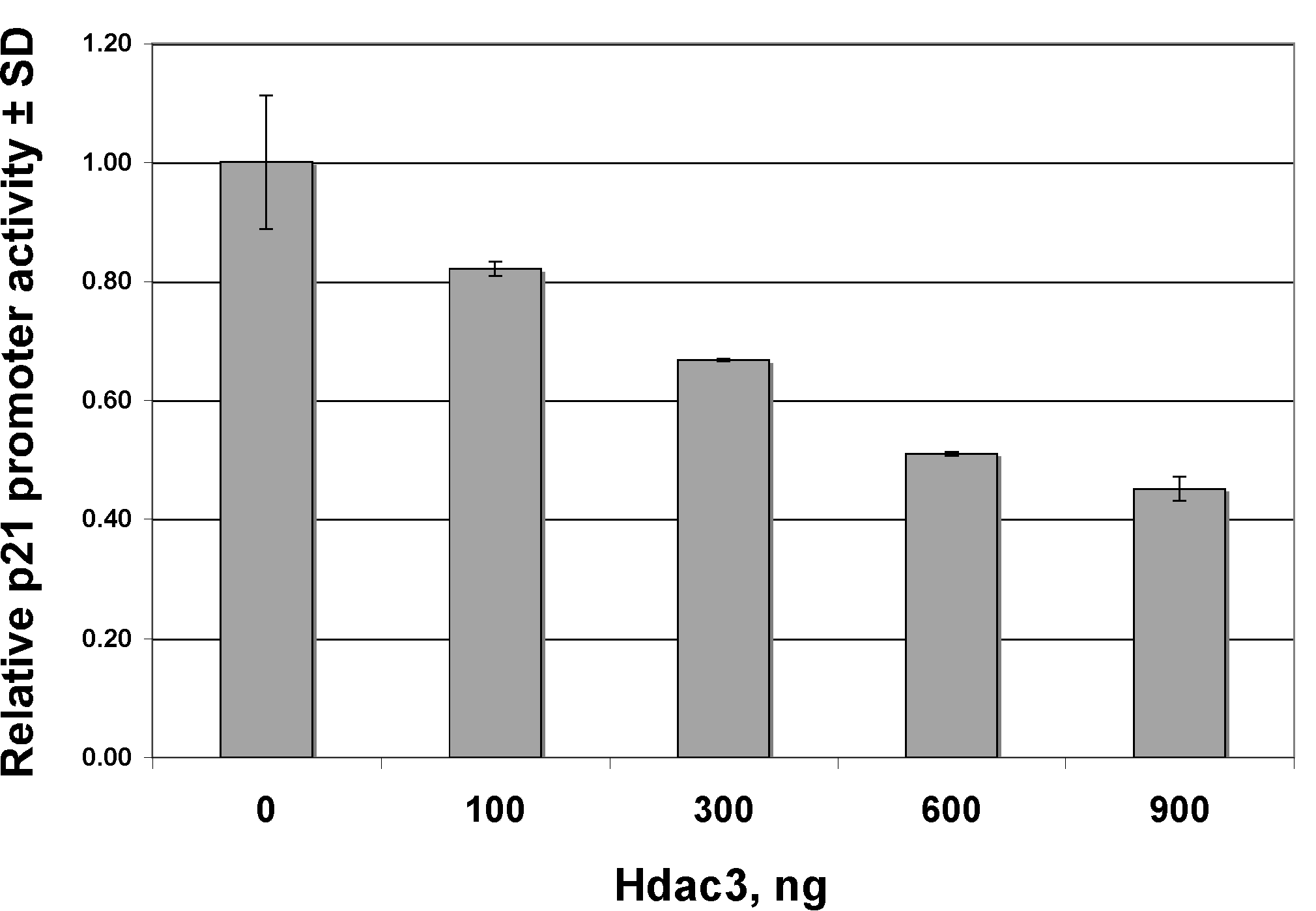

Supplement: Figure S4 — HDAC3 represses the p21(CIP1) promoter. C2C12 cells were transfected with a p21-luciferase reporter and increasing concentrations of pCMV-HDAC3. Firefly luciferase activity was normalized to renilla-luciferase activity to control for transfection efficiency. Data are shown relative to cells transfected with an empty CMV vector. (0.04 MB TIF) [file pone.0011492.s004.tif]

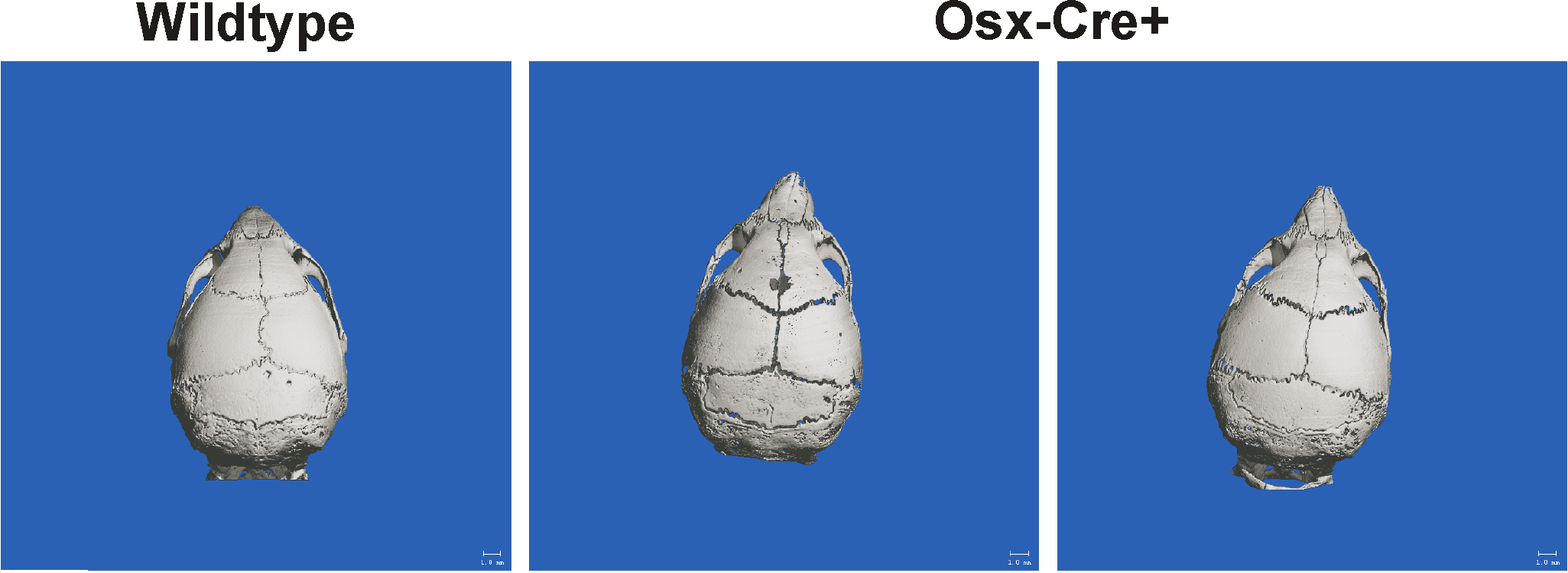

Supplement: Figure S5 — MicroCT Reconstructions of skulls from Osx-Cre mice. Minor craniofacial defects are present in 5.5 week-old Osx-Cre mice. These mice are wildtype at the Hdac3 loci. (0.35 MB TIF) [file pone.0011492.s005.tif]
