# Supplementary material for: Histone Deacetylase 3 Depletion in Osteo/Chondroprogenitor Cells Decreases Bone Density and Increases Marrow Fat
Source: PLoS One. 2010 Jul 9;5(7):e11492. doi: 10.1371/journal.pone.0011492 (PMC2901996; doi:10.1371/journal.pone.0011492)
Supplement: Table S1 — List of the 176 genes common to two independent microarray experiments. (0.14 MB PDF) [file pone.0011492.s006.pdf]

	A	B	C	D	E	F	G	H	I
1	SYMBOL	Mean(KO)_exp1	Mean(WT)_exp1	log2ratio(KOvsWT)_exp1	p-value(WT vs. KO)_exp1	Fold-Change(KO/WT)_exp1	Mean(KO)_exp2	Mean(WT)_exp2	log2ratio_exp2
2	Cdkn1a	11.774	10.0232	1.7508	0.000488906	3.36548	11.1188	9.36188	1.75692
3	Ephx1	8.53408	7.10818	1.4259	0.000456095	2.68681	8.24687	6.72974	1.51713
4	Plat	8.76877	7.89959	0.86918	0.000480702	1.82662	8.66719	7.54744	1.11975
5	Icam1	9.7452	9.19917	0.54603	0.00608673	1.46006	9.99299	8.89155	1.10144
6	AB023957	7.43848	6.61673	0.82175	0.0310754	1.76754	8.2552	7.16946	1.08574
7	Phlda3	9.00871	8.06815	0.94056	0.00239852	1.91928	9.00543	7.91998	1.08545
8	Nrp1	10.4152	9.67555	0.73965	0.0140421	1.6698	10.4223	9.45406	0.96824
9	Tmem178	7.24804	6.25835	0.98969	0.00564331	1.98577	7.38597	6.43637	0.9496
10	Gpnmb	7.51083	6.99276	0.51807	0.0352094	1.43204	7.42737	6.52208	0.90529
11	Mmp2	13.8509	13.3854	0.4655	0.0242932	1.38077	13.7775	12.8727	0.9048
12	Tnfrsf11b	7.33113	6.97361	0.35752	0.0133059	1.28123	7.70677	6.84115	0.86562
13	Gas6	10.622	10.0132	0.6088	0.0189933	1.52499	10.5818	9.77355	0.80825
14	Slc19a2	7.58158	6.92847	0.65311	0.00207923	1.57255	7.99097	7.20408	0.78689
15	Tmem43	10.1441	9.45083	0.69327	0.000215618	1.617	10.2433	9.46452	0.77878
16	Anxa8	7.30089	6.64108	0.65981	0.00043334	1.57988	7.24455	6.48302	0.76153
17	Slc29a3	10.6333	10.1215	0.5118	0.00329876	1.4258	10.8002	10.0416	0.7586
18	Timp3	10.5251	9.95367	0.57143	0.0267644	1.48595	10.2025	9.44949	0.75301
19	LOC10004(	6.72183	6.02282	0.69901	0.0228087	1.62338	6.88789	6.14004	0.74785
20	Slc16a9	8.9059	8.36263	0.54327	0.0189925	1.45726	9.17071	8.42739	0.74332
21	Gstm6	7.55378	6.44888	1.1049	0.00209928	2.15084	6.90638	6.1653	0.74108
22	Aaas	7.77148	7.12704	0.64444	0.00026165	1.56313	7.32957	6.60045	0.72912
23	Tpm1	10.9346	10.6003	0.3343	0.0174136	1.26079	10.939	10.2118	0.7272
24	Ahnak2	7.80684	7.17832	0.62852	0.0223786	1.54598	7.76862	7.04438	0.72424
25	Exoc4	7.54025	7.10736	0.43289	0.00785566	1.34994	7.98939	7.26703	0.72236
26	Lgr5	6.44579	5.90427	0.54152	0.00506022	1.45551	6.92361	6.20376	0.71985
27	E430002G(	8.16314	7.41559	0.74755	0.00342105	1.67894	8.7768	8.07762	0.69918
28	Prelp	11.2492	10.7219	0.5273	0.0498294	1.44118	11.6809	11.003	0.6779
29	Palld	12.3025	11.924	0.3785	0.00518872	1.30003	12.4378	11.7625	0.6753
30	LOC100047	9.81256	9.16506	0.6475	0.014647	1.56645	9.17456	8.50262	0.67194
31	Bcl11b	8.93913	8.3965	0.54263	0.00297636	1.45663	9.41025	8.75379	0.65646
32	Hmgcs2	7.11809	6.79347	0.32462	0.0119446	1.25233	7.57408	6.92062	0.65346
33	Scd2	12.3986	11.8344	0.5642	0.00628111	1.47859	12.5899	11.9422	0.6477
34	Polk	7.57054	7.21106	0.35948	0.0311492	1.28297	7.76825	7.14834	0.61991

	A	B	C	D	E	F	G	H	I
1	**SYMBOL**	**Mean(KO)_exp1**	**Mean(WT)_exp1**	**log2ratio(KOvsWT)_exp1**	**p-value(WT vs. KO)_exp1**	**Fold-Change(KO/WT)_exp1**	**Mean(KO)_exp2**	**Mean(WT)_exp2**	**log2ratio_exp2**
35	Lgals3bp	8.68509	8.19617	0.48892	0.0172717	1.40339	7.96954	7.35312	0.61642
36	Angptl4	9.73073	9.02142	0.70931	0.0386718	1.63502	9.57485	8.97527	0.59958
37	6330406I15	7.39563	6.66961	0.72602	0.0366175	1.65407	7.775	7.18118	0.59382
38	Pax1	8.11415	7.28067	0.83348	0.014363	1.78198	7.9054	7.31275	0.59265
39	Cxcl2	7.90026	7.31359	0.58667	0.0191929	1.50178	8.02404	7.43681	0.58723
40	Ednra	10.7144	10.569	0.1454	0.010835	1.106	10.9192	10.3381	0.5811
41	Sfrp4	8.05608	7.55131	0.50477	0.0330263	1.4189	8.44706	7.89222	0.55484
42	Eef2	10.8943	10.7252	0.1691	0.0150659	1.12438	10.9488	10.4097	0.5391
43	Atp1b1	9.29991	9.20399	0.09592	0.00255164	1.06874	9.67722	9.13975	0.53747
44	Ror2	8.6428	8.09016	0.55264	0.0360003	1.46677	8.59683	8.06356	0.53327
45	Thbs2	13.2907	13.0216	0.2691	0.0100208	1.20508	13.5055	12.9743	0.5312
46	Rnf145	8.48214	8.10145	0.38069	0.00105113	1.30197	8.4124	7.8841	0.5283
47	Igfbp4	12.11	11.8002	0.3098	0.00738228	1.23954	12.3694	11.8423	0.5271
48	Kcne4	8.56435	8.23175	0.3326	0.0184764	1.25928	8.36011	7.8499	0.51021
49	Col5a1	13.8306	13.2831	0.5475	0.0208599	1.4616	13.6771	13.1737	0.5034
50	B4galt1	9.05173	8.80937	0.24236	0.0376083	1.18293	9.01581	8.51511	0.5007
51	Ccnd1	9.69774	9.09075	0.60699	0.0154819	1.52308	9.64448	9.14567	0.49881
52	Fzd7	7.28962	7.11918	0.17044	0.0227966	1.1254	7.65494	7.15651	0.49843
53	Axin2	6.42735	5.98069	0.44666	0.0262626	1.36289	6.61005	6.11875	0.4913
54	Cd14	8.21723	7.48441	0.73282	0.0411215	1.66188	7.69608	7.21216	0.48392
55	Plp1	6.47036	5.97652	0.49384	0.0284665	1.40819	6.848	6.36518	0.48282
56	Emilin1	9.76253	9.36873	0.3938	0.045256	1.31384	9.82248	9.34086	0.48162
57	Angptl2	9.05251	8.36652	0.68599	0.0338137	1.60881	8.56176	8.08045	0.48131
58	Dpep1	7.63353	7.01547	0.61806	0.019549	1.53482	7.54122	7.06286	0.47836
59	Cacna1g	7.63258	7.18273	0.44985	0.0246323	1.3659	8.05814	7.59331	0.46483
60	Sgms2	9.81974	9.32823	0.49151	0.0200425	1.40591	9.17259	8.71004	0.46255
61	Nfatc4	9.90726	9.73926	0.168	0.0137744	1.1235	10.1307	9.66997	0.46073
62	Mapkapk3	6.88574	6.35563	0.53011	0.0119302	1.44404	6.8009	6.34021	0.46069
63	Lmna	11.6745	11.2313	0.4432	0.00846788	1.35962	11.7033	11.245	0.4583
64	Mdm2	8.76264	8.48045	0.28219	0.0254071	1.21605	9.14069	8.68667	0.45402
65	Il1rl1	6.61948	6.23044	0.38904	0.000213572	1.30952	6.51401	6.06165	0.45236
66	Vcl	10.6462	10.3276	0.3186	0.0118168	1.24712	10.4537	10.0116	0.4421
67	Mrgprf	8.47731	7.68883	0.78848	0.0120274	1.72727	8.12447	7.68546	0.43901

	A	B	C	D	E	F	G	H	I
1	SYMBOL	Mean(KO)_exp1	Mean(WT)_exp1	log2ratio(KOvsWT)_exp1	p-value(WT vs. KO)_exp1	Fold-Change(KO/WT)_exp1	Mean(KO)_exp2	Mean(WT)_exp2	log2ratio_exp2
68	Ctsd	10.0253	9.8452	0.1801	0.0355033	1.13295	9.63929	9.20156	0.43773
69	Itga11	10.7075	10.319	0.3885	0.0414586	1.30903	10.7056	10.2805	0.4251
70	Klf9	10.6409	10.2087	0.4322	0.0443582	1.34922	10.5246	10.1034	0.4212
71	Fzd1	7.26073	6.87771	0.38302	0.0183537	1.30407	8.50941	8.09534	0.41407
72	Aebp1	12.6971	12.4299	0.2672	0.0153557	1.20347	12.6549	12.241	0.4139
73	Dclk1	6.8877	6.45688	0.43082	0.0222223	1.348	7.0948	6.68539	0.40941
74	Klhl22	8.45376	8.20957	0.24419	0.0352844	1.18443	8.54259	8.13483	0.40776
75	Pappa	6.54025	6.22334	0.31691	0.0322242	1.24566	6.66132	6.25376	0.40756
76	Clip1	7.10869	6.84212	0.26657	0.015744	1.20295	7.2277	6.82094	0.40676
77	Sel1l	8.9966	8.77199	0.22461	0.00982749	1.16847	9.34867	8.94729	0.40138
78	Fbn1	9.71171	9.41568	0.29603	0.0307088	1.22776	10.2658	9.86721	0.39859
79	Gria3	6.46537	6.20704	0.25833	0.0067918	1.1961	6.4974	6.10409	0.39331
80	Scd1	8.86547	8.33253	0.53294	0.0147557	1.44688	8.67608	8.28445	0.39163
81	Sqstm1	9.96434	9.63577	0.32857	0.0244607	1.25577	10.1159	9.73006	0.38584
82	Lgi2	8.16755	7.55322	0.61433	0.00290713	1.53085	8.14844	7.76322	0.38522
83	LOC10004!	6.94412	6.59481	0.34931	0.0292268	1.27394	6.80174	6.42535	0.37639
84	Tmem132c	6.5419	6.08363	0.45827	0.0172257	1.37389	6.57759	6.21385	0.36374
85	Trim11	7.42684	7.25141	0.17543	0.0407933	1.1293	7.46722	7.11164	0.35558
86	Eno2	7.40363	6.94181	0.46182	0.00471241	1.37728	7.50748	7.18661	0.32087
87	Zfp516	6.73291	6.58429	0.14862	0.0487243	1.10851	6.86602	6.56228	0.30374
88	Car12	6.61415	6.33807	0.27608	0.0160248	1.2109	6.64445	6.34366	0.30079
89	Slc1a4	9.81086	9.4217	0.38916	0.0381077	1.30963	9.88734	9.58666	0.30068
90	Aox1	6.34727	6.06734	0.27993	0.0255057	1.21414	6.37353	6.07643	0.2971
91	Rab11fip5	9.3288	9.21955	0.10925	0.0341536	1.07867	9.36513	9.07765	0.28748
92	Coq10b	8.69067	8.36602	0.32465	0.0467361	1.25236	8.21611	7.93234	0.28377
93	Zfp532	7.00878	6.91087	0.09791	0.0301438	1.07022	6.94999	6.66764	0.28235
94	Tbc1d22a	8.81595	8.69652	0.11943	0.00253415	1.08631	9.38126	9.112	0.26926
95	Fasn	10.3237	9.95817	0.36553	0.013545	1.2884	10.6114	10.3467	0.2647
96	Cachd1	6.40214	6.24022	0.16192	0.0235586	1.11878	6.68801	6.44031	0.2477
97	Tcn2	8.19656	8.49347	-0.29691	0.0174446	-1.22851	8.17604	7.94044	0.2356
98	LOC10004!	7.50942	7.09117	0.41825	0.0260863	1.33631	7.49709	7.26261	0.23448
99	Kcns1	7.08502	6.68951	0.39551	0.00726877	1.31541	6.94286	6.71239	0.23047
100	Ncoa5	7.9421	7.78743	0.15467	0.0238044	1.11317	8.32148	8.09648	0.225

	A	B	C	D	E	F	G	H	I
1	**SYMBOL**	**Mean(KO)_exp1**	**Mean(WT)_exp1**	**log2ratio(KOvsWT)_exp1**	**p-value(WT vs. KO)_exp1**	**Fold-Change(KO/WT)_exp1**	**Mean(KO)_exp2**	**Mean(WT)_exp2**	**log2ratio_exp2**
101	Il11ra1	12.5866	12.393	0.1936	0.0353914	1.14369	12.9159	12.7261	0.1898
102	Sars	7.86601	7.65479	0.21122	0.00392972	1.15767	7.64195	7.4545	0.18745
103	Rab15	8.77621	8.50424	0.27197	0.012921	1.20746	9.19848	9.01884	0.17964
104	Ppap2a	8.78336	8.51541	0.26795	0.037513	1.20409	8.6782	8.50313	0.17507
105	Gle1	6.93646	6.82793	0.10853	0.02121	1.07813	6.88575	6.73197	0.15378
106	Snrp70	7.16649	7.38941	-0.22292	0.0274654	-1.16709	7.02215	6.8768	0.14535
107	Zfp295	6.14386	6.03603	0.10783	0.0195411	1.07761	6.09183	5.9491	0.14273
108	Dhcr24	6.21452	6.0608	0.15372	0.0337092	1.11243	6.15693	6.03364	0.12329
109	Nr1h2	6.81726	6.94683	-0.12957	0.0042496	-1.09396	6.84476	6.73476	0.11
110	Vamp4	8.46574	8.73764	-0.2719	0.00900001	-1.2074	8.67627	8.57769	0.09858
111	Sts	6.23119	6.01565	0.21554	0.0309721	1.16114	5.9923	6.05072	-0.05842
112	Gpm6a	5.9111	5.98478	-0.07368	0.0164849	-1.0524	5.78572	5.84671	-0.06099
113	Rnf167	7.71143	7.9111	-0.19967	0.0214377	-1.14843	7.67717	7.76189	-0.08472
114	LOC10004[illegible]	7.24325	7.52749	-0.28424	0.0311643	-1.21777	7.29211	7.39585	-0.10374
115	OTTMUSG	5.99283	6.16763	-0.1748	0.0196245	-1.12881	6.07248	6.18357	-0.11109
116	LOC67622[illegible]	6.01247	6.11527	-0.1028	0.0369157	-1.07386	5.86144	5.9794	-0.11796
117	EG432982	6.14214	6.28327	-0.14113	0.0160637	-1.10277	6.02541	6.15711	-0.1317
118	Slc29a1	8.48814	8.95028	-0.46214	0.0466004	-1.37758	8.25377	8.39831	-0.14454
119	Ccbp2	6.35363	6.69314	-0.33951	0.0490006	-1.26533	6.18272	6.33434	-0.15162
120	AA467197	6.13156	6.32714	-0.19558	0.0286261	-1.14518	5.94051	6.11247	-0.17196
121	Bbs12	6.40323	6.57816	-0.17493	0.0119832	-1.12891	6.37713	6.55528	-0.17815
122	Rpusd4	7.68332	7.83504	-0.15172	0.0428885	-1.1109	7.99592	8.19265	-0.19673
123	Tmem159	7.4924	7.70935	-0.21695	0.0146168	-1.16227	7.54403	7.74109	-0.19706
124	Bcas3	9.70087	9.99109	-0.29022	0.0200827	-1.22283	9.8132	10.0261	-0.2129
125	Cib3	5.83739	6.12057	-0.28318	0.0273138	-1.21688	5.7747	5.99809	-0.22339
126	Cr1l	6.62756	7.03906	-0.4115	0.0140681	-1.33007	6.92056	7.1528	-0.23224
127	9330154J0[illegible]	6.346	6.63498	-0.28898	0.0256881	-1.22177	6.25539	6.49105	-0.23566
128	5730403B1	7.61842	7.88442	-0.266	0.00615409	-1.20246	7.19922	7.43721	-0.23799
129	LOC10004[illegible]	6.56285	6.94499	-0.38214	0.0430746	-1.30328	6.46835	6.73077	-0.26242
130	Ncald	6.96412	7.21009	-0.24597	0.00172599	-1.1859	7.35939	7.62183	-0.26244
131	H2-T10	6.61907	6.87405	-0.25498	0.0218153	-1.19332	6.29225	6.56159	-0.26934
132	Gimap1	5.85792	6.00311	-0.14519	0.00281777	-1.10587	5.72887	6.01326	-0.28439
133	Arid5b	5.95298	5.83436	0.11862	0.0156498	1.08569	5.81551	6.10142	-0.28591

	A	B	C	D	E	F	G	H	I
1	**SYMBOL**	**Mean(KO)_exp1**	**Mean(WT)_exp1**	**log2ratio(KOvsWT)_exp1**	**p-value(WT vs. KO)_exp1**	**Fold-Change(KO/WT)_exp1**	**Mean(KO)_exp2**	**Mean(WT)_exp2**	**log2ratio_exp2**
134	Fahd2a	6.37975	6.59458	-0.21483	0.00131395	-1.16058	6.16769	6.45929	-0.2916
135	Ctsw	6.22623	6.78592	-0.55969	0.0108038	-1.47395	5.75115	6.05458	-0.30343
136	Sytl4	6.69992	7.07951	-0.37959	0.0335988	-1.30097	6.56936	6.8732	-0.30384
137	Rbm11	6.00737	6.21656	-0.20919	0.0111039	-1.15604	5.9265	6.23538	-0.30888
138	Cd200	8.08096	8.76116	-0.6802	0.00142161	-1.60237	8.13535	8.45118	-0.31583
139	8430408G2	6.328	6.89071	-0.56271	0.0362379	-1.47704	6.1292	6.45975	-0.33055
140	Gper	7.0049	7.48713	-0.48223	0.012422	-1.39691	7.0421	7.37864	-0.33654
141	Rhbdl3	6.22329	6.47405	-0.25076	0.0387761	-1.18984	5.98986	6.34642	-0.35656
142	Ccdc3	11.2287	11.8974	-0.6687	0.00399925	-1.58969	11.3198	11.6809	-0.3611
143	Prdx4	11.8267	12.0829	-0.2562	0.0415112	-1.19433	11.8212	12.1845	-0.3633
144	Nqo2	6.85409	7.17569	-0.3216	0.00726951	-1.24972	6.68341	7.05173	-0.36832
145	Sort1	7.82098	8.22948	-0.4085	0.0147834	-1.32731	7.88721	8.25834	-0.37113
146	Pfkl	9.01056	9.32901	-0.31845	0.00815908	-1.24699	8.81376	9.18569	-0.37193
147	Dapk2	6.99984	7.37137	-0.37153	0.00179676	-1.29372	6.79814	7.18109	-0.38295
148	Adam12	6.50119	6.94207	-0.44088	0.0118003	-1.35743	6.62783	7.02118	-0.39335
149	Wif1	9.62521	10.1892	-0.56399	0.0266657	-1.4784	9.93766	10.3333	-0.39564
150	Cxcl12	9.04152	9.57983	-0.53831	0.0435781	-1.45227	9.35234	9.74877	-0.39643
151	Dner	7.80442	8.44144	-0.63702	0.000864277	-1.55511	8.18963	8.5972	-0.40757
152	2310005E1	7.12587	7.39604	-0.27017	0.0367852	-1.20594	6.81121	7.239	-0.42779
153	St3gal6	8.47981	9.16026	-0.68045	0.0181543	-1.60264	8.41924	8.8631	-0.44386
154	Ranbp3l	6.6712	7.58412	-0.91292	0.0144707	-1.88286	6.98289	7.45112	-0.46823
155	Gfra1	6.82775	7.47475	-0.647	0.00666123	-1.5659	6.98846	7.46242	-0.47396
156	Prr15	7.27853	7.95423	-0.6757	0.0117969	-1.59737	7.19272	7.69694	-0.50422
157	Cdk5r1	7.26451	7.85117	-0.58666	0.00200548	-1.50176	7.44875	8.02529	-0.57654
158	Hdac3	8.28298	9.54559	-1.26261	0.00383398	-2.3993	9.12609	9.71985	-0.59376
159	Cmbl	6.15889	6.40181	-0.24292	0.0299817	-1.18339	6.1345	6.72966	-0.59516
160	Vit	7.80333	9.02659	-1.22326	0.0311736	-2.33474	8.23814	8.83641	-0.59827
161	Rbp7	6.13913	6.386	-0.24687	0.0395521	-1.18664	6.0087	6.61723	-0.60853
162	Ndufa1	11.4888	11.8619	-0.3731	0.0412521	-1.2952	11.1637	11.7744	-0.6107
163	Hey1	7.88567	8.62942	-0.74375	0.0105763	-1.67452	7.51808	8.15097	-0.63289
164	Ankrd37	6.15846	6.72737	-0.56891	0.0233644	-1.4834	6.03479	6.66876	-0.63397
165	Hpgd	8.6545	9.64361	-0.98911	0.000998965	-1.98496	8.89629	9.57069	-0.6744
166	Cd59b	6.71051	7.37713	-0.66662	0.00694943	-1.58736	6.61842	7.2931	-0.67468

	A	B	C	D	E	F	G	H	I
1	**SYMBOL**	**Mean(KO)_exp1**	**Mean(WT)_exp1**	**log2ratio(KOvsWT)_exp1**	**p-value(WT vs. KO)_exp1**	**Fold-Change(KO/WT)_exp1**	**Mean(KO)_exp2**	**Mean(WT)_exp2**	**log2ratio_exp2**
167	Icam2	7.47721	8.09953	-0.62232	0.00244788	-1.53934	7.72724	8.40347	-0.67623
168	Serpina3g	7.00826	8.19119	-1.18293	0.0341934	-2.27039	7.27912	7.97434	-0.69522
169	Gpihbp1	8.18426	8.78083	-0.59657	0.0408673	-1.51212	8.07534	8.86071	-0.78537
170	Entpd3	10.6653	11.4316	-0.7663	0.00226235	-1.70096	10.3858	11.1719	-0.7861
171	Cd59a	8.97001	9.53288	-0.56287	0.0182944	-1.47721	8.93431	9.91052	-0.97621
172	Sct	6.05268	6.42118	-0.3685	0.0329781	-1.29101	6.0067	6.98379	-0.97709
173	Mepe	7.84893	8.78899	-0.94006	0.00377094	-1.91861	6.90969	7.99804	-1.08835
174	Rsad2	6.79095	7.97154	-1.18059	0.00934478	-2.26669	6.81371	7.91135	-1.09764
175	Alas2	7.34878	8.92497	-1.57619	0.0407246	-2.98182	7.64422	8.7672	-1.12298
176	Ramp1	8.32506	9.33727	-1.01221	0.000460241	-2.017	7.98986	9.36527	-1.37541
177	S100a8	8.32825	10.7332	-2.40495	0.0427049	-5.29635	7.75036	10.9787	-3.22834

	J	K	L	M	N	O	P	Q	R	S
1	p-value(KO vs. WT)_exp2	FDR(p-value(KO vs. WT))_exp2	Fold-Change(KO vs. WT)_exp2	FC>1SD	FC>2SD	FC>3SD	CHROMOSOME	CYTOBAND	ENTREZ_GENE_ID	ILMN_GENE
2	0.00715871	0.526724	3.37979	TRUE	TRUE	TRUE	17	17qA3.3	12575	CDKN1A
3	0.000560381	0.509961	2.86222	TRUE	TRUE	TRUE	1	1qH4	13849	EPHX1
4	0.004289	0.516318	2.1731	TRUE	TRUE	TRUE				PLAT
5	0.0489971	0.561001	2.14568	TRUE	TRUE	FALSE	9	9qA3	15894	ICAM1
6	0.00230393	0.516318	2.12246	TRUE	TRUE	TRUE				AB023957
7	0.016333	0.538897	2.12203	TRUE	TRUE	TRUE	1	1qE4	27280	PHLDA3
8	0.0305405	0.553513	1.9565	TRUE	TRUE	TRUE	8	8qE2	18186	NRP1
9	0.00577256	0.516318	1.93134	TRUE	TRUE	TRUE	17	17qE3	68027	TMEM178
10	0.0139083	0.538897	1.87293	TRUE	TRUE	FALSE	6	6qB2.3	93695	GPNMB
11	0.0196987	0.538897	1.87232	TRUE	TRUE	FALSE	8	8qC5	17390	MMP2
12	0.0109191	0.538897	1.82212	TRUE	FALSE	FALSE	15	15qD1	18383	TNFRSF11
13	0.0135283	0.538897	1.75106	TRUE	TRUE	FALSE		8qA1.1	14456	GAS6
14	0.000343565	0.509961	1.72535	TRUE	TRUE	FALSE	1	1qH2.2	116914	SLC19A2
15	0.00293133	0.516318	1.7157	TRUE	TRUE	FALSE	6	6qD1	74122	TMEM43
16	0.0392052	0.559686	1.69529	TRUE	TRUE	FALSE	14	14qB	11752	ANXA8
17	0.0215248	0.538897	1.69185	TRUE	TRUE	FALSE	10	10qB4	71279	SLC29A3
18	0.00667087	0.526724	1.68527	TRUE	TRUE	FALSE	10	10qC1	21859	TIMP3
19	0.00132607	0.516318	1.67929	TRUE	TRUE	FALSE			1E+08	LOC100046
20	0.0172062	0.538897	1.67403	TRUE	TRUE	FALSE		10qB5.3	66859	SLC16A9
21	0.0301927	0.553513	1.67143	TRUE	TRUE	FALSE	3	3qF2.3	14867	GSTM6
22	0.02275	0.541998	1.65763	TRUE	TRUE	FALSE	15	15qF3	223921	AAAS
23	0.0317857	0.553513	1.65541	TRUE	FALSE	FALSE		9qC	22003	TPM1
24	0.0103278	0.538897	1.65203	TRUE	TRUE	FALSE	12	12qF1	382643	AHNAK2
25	0.00517615	0.516318	1.64988	TRUE	TRUE	FALSE	6	6qA3.3	20336	EXOC4
26	0.013171	0.538897	1.647	TRUE	TRUE	FALSE	10	10qD2	14160	LGR5
27	0.0179439	0.538897	1.62358	TRUE	TRUE	FALSE				E430002G0
28	0.0164665	0.538897	1.5998	TRUE	TRUE	FALSE	1	1qE4	116847	PRELP
29	0.0216367	0.538897	1.59701	TRUE	TRUE	FALSE	8	8qB3.1	72333	PALLD
30	0.0499315	0.561001	1.59321	TRUE	TRUE	FALSE			1E+08	LOC100047
31	0.0114724	0.538897	1.57621	TRUE	TRUE	FALSE	12	12qF1	58208	BCL11B
32	0.0208177	0.538897	1.57294	TRUE	FALSE	FALSE	3	3qF2.2	15360	HMGCS2
33	0.00579911	0.516318	1.56666	TRUE	TRUE	FALSE	19	19qC3	20250	SCD2
34	0.00569741	0.516318	1.53678	TRUE	FALSE	FALSE	13	13qD1	27015	POLK

	J	K	L	M	N	O	P	Q	R	S
1	p-value(KO vs. WT)_exp2	FDR(p-value(KO vs. WT))_exp2	Fold-Change(KO vs. WT)_exp2	FC>1SD	FC>2SD	FC>3SD	CHROMOSOME	CYTOBAND	ENTREZ_GENE_ID	ILMN_GENE
35	0.00458751	0.516318	1.53306	TRUE	TRUE	FALSE	11	11qE2	19039	LGALS3BP
36	0.026973	0.550491	1.51528	TRUE	TRUE	FALSE				ANGPTL4
37	0.0442174	0.561001	1.50923	TRUE	TRUE	FALSE		5qG3	70717	6330406I15
38	0.00307893	0.516318	1.50802	TRUE	TRUE	FALSE	2	2qG2	18503	PAX1
39	0.0158684	0.538897	1.50236	TRUE	TRUE	FALSE				CXCL2
40	0.035878	0.554215	1.49594	FALSE	FALSE	FALSE	8	8qC1	13617	EDNRA
41	0.0438276	0.561001	1.46901	TRUE	FALSE	FALSE	13	13qA2	20379	SFRP4
42	0.0434642	0.561001	1.45307	FALSE	FALSE	FALSE	10	10qC1	13629	EEF2
43	0.0389256	0.559338	1.45143	FALSE	FALSE	FALSE		1qH2.2	11931	ATP1B1
44	0.0160864	0.538897	1.4472	TRUE	FALSE	FALSE	13	13qB1	26564	ROR2
45	0.044801	0.561001	1.44516	TRUE	FALSE	FALSE	17	17qA2	21826	THBS2
46	0.0282653	0.550491	1.44223	TRUE	FALSE	FALSE	11	11qB1.1	74315	RNF145
47	0.0017075	0.516318	1.44101	TRUE	FALSE	FALSE	11	11qD	16010	IGFBP4
48	0.0320138	0.553513	1.42426	TRUE	FALSE	FALSE	1	1qC4	57814	KCNE4
49	0.0199453	0.538897	1.41753	TRUE	FALSE	FALSE	2	2qA3	12831	COL5A1
50	0.00934591	0.538897	1.4149	TRUE	FALSE	FALSE	4	4qA5	14595	B4GALT1
51	0.0187568	0.538897	1.41305	TRUE	FALSE	FALSE	7	7qF5	12443	CCND1
52	0.000747127	0.509961	1.41268	FALSE	FALSE	FALSE	1	1qC1.3	14369	FZD7
53	0.0323077	0.553513	1.40571	TRUE	FALSE	FALSE	11	11qE1	12006	AXIN2
54	0.0135134	0.538897	1.39854	TRUE	FALSE	FALSE	18	18qB2	12475	CD14
55	0.025233	0.550491	1.39748	TRUE	FALSE	FALSE	X	XqF1	18823	PLP1
56	0.0179843	0.538897	1.39631	TRUE	FALSE	FALSE	5	5qB1	100952	EMILIN1
57	0.0329883	0.553513	1.39601	TRUE	FALSE	FALSE	2	2qB	26360	ANGPTL2
58	0.0323454	0.553513	1.39315	TRUE	FALSE	FALSE	8	8qE1	13479	DPEP1
59	0.00605563	0.516318	1.38016	TRUE	FALSE	FALSE	11	11qD	12291	CACNA1G
60	0.0327037	0.553513	1.37797	TRUE	FALSE	FALSE		3qG3	74442	SGMS2
61	0.00833744	0.538456	1.37624	FALSE	FALSE	FALSE	14	14qC3	73181	NFATC4
62	0.0267373	0.550491	1.3762	TRUE	FALSE	FALSE				MAPKAPK3
63	0.0406852	0.559731	1.37394	TRUE	FALSE	FALSE	3	3qF1	16905	LMNA
64	0.040279	0.559731	1.36985	TRUE	FALSE	FALSE	10	10qD2	17246	MDM2
65	0.00300294	0.516318	1.36828	TRUE	FALSE	FALSE	1	1qB	17082	IL1RL1
66	0.0194233	0.538897	1.35858	TRUE	FALSE	FALSE	14	14qA3	22330	VCL
67	0.049069	0.561001	1.35568	TRUE	FALSE	FALSE	7	7qF5	211577	MRGPRF

	J	K	L	M	N	O	P	Q	R	S
1	p-value(KO vs. WT)_exp2	FDR(p-value(KO vs. WT))_exp2	Fold-Change(KO vs. WT)_exp2	FC>1SD	FC>2SD	FC>3SD	CHROMOSOME	CYTOBAND	ENTREZ_GENE_ID	ILMN_GENE
68	0.0146076	0.538897	1.35448	FALSE	FALSE	FALSE	7	7qF5	13033	CTSD
69	0.0412588	0.559731	1.34266	TRUE	FALSE	FALSE	9	9qB	319480	ITGA11
70	0.0368359	0.554215	1.3391	TRUE	FALSE	FALSE		19qB	16601	KLF9
71	0.0225729	0.5418	1.33244	TRUE	FALSE	FALSE	5	5qA1	14362	FZD1
72	0.0395158	0.559731	1.33225	TRUE	FALSE	FALSE	11	11qA1	11568	AEBP1
73	0.0367327	0.554215	1.32814	TRUE	FALSE	FALSE	3	3qC	13175	DCLK1
74	0.0151515	0.538897	1.32662	TRUE	FALSE	FALSE	16	16qA3	224023	KLHL22
75	0.0223753	0.54062	1.32644	TRUE	FALSE	FALSE	4	4qC1	18491	PAPPA
76	0.0413566	0.559731	1.3257	TRUE	FALSE	FALSE		5qF	56430	CLIP1
77	0.0488547	0.561001	1.32077	TRUE	FALSE	FALSE	12	12qD3	20338	SEL1L
78	0.028268	0.550491	1.31821	TRUE	FALSE	FALSE	2	2qF1	14118	FBN1
79	0.00624611	0.516318	1.3134	TRUE	FALSE	FALSE	X	XqA3.3-qA4	53623	GRIA3
80	0.0281472	0.550491	1.31188	TRUE	FALSE	FALSE	19	19qC3	20249	SCD1
81	0.0341441	0.554215	1.30665	TRUE	FALSE	FALSE	11	11qB1.3	18412	SQSTM1
82	0.0329275	0.553513	1.30606	TRUE	FALSE	FALSE	5	5qC1	246316	LGI2
83	0.0492734	0.561001	1.29808	TRUE	FALSE	FALSE			1E+08	LOC100045
84	0.0134865	0.538897	1.28676	TRUE	FALSE	FALSE		5qG1.2	208213	TMEM132C
85	0.00719799	0.526724	1.27949	FALSE	FALSE	FALSE	11	11qB1.3	94091	TRIM11
86	0.0485795	0.561001	1.24908	TRUE	FALSE	FALSE	6	6qF2	13807	ENO2
87	0.048232	0.561001	1.23434	FALSE	FALSE	FALSE	18	18qE3	329003	ZFP516
88	0.00357038	0.516318	1.23182	TRUE	FALSE	FALSE				CAR12
89	0.010622	0.538897	1.23173	TRUE	FALSE	FALSE	11	11qA3.1	55963	SLC1A4
90	0.0207888	0.538897	1.22867	TRUE	FALSE	FALSE	1	1qC1.3	11761	AOX1
91	0.0189763	0.538897	1.22051	FALSE	FALSE	FALSE	6	6qC3	52055	RAB11FIP5
92	0.00969765	0.538897	1.21737	FALSE	FALSE	FALSE	1	1qC1.2	67876	COQ10B
93	0.0274262	0.550491	1.21618	FALSE	FALSE	FALSE	18	18qE1	328977	ZFP532
94	0.027494	0.550491	1.20519	FALSE	FALSE	FALSE	15	15qE2	223754	TBC1D22A
95	0.0464932	0.561001	1.20141	FALSE	FALSE	FALSE	11	11qE2	14104	FASN
96	0.0266576	0.550491	1.18732	FALSE	FALSE	FALSE	4	4qC6	320508	CACHD1
97	0.0311764	0.553513	1.17739	FALSE	FALSE	FALSE	11	11qA1	21452	TCN2
98	0.0351795	0.554215	1.17648	FALSE	FALSE	FALSE			1E+08	LOC100045
99	0.0300486	0.553513	1.17322	FALSE	FALSE	FALSE	2	2qH3	16538	KCNS1
100	0.0162302	0.538897	1.16877	FALSE	FALSE	FALSE	2	2qH3	228869	NCOA5

	J	K	L	M	N	O	P	Q	R	S
1	**p-value(KO vs. WT)_exp2**	**FDR(p-value(KO vs. WT))_exp2**	**Fold-Change(KO vs. WT)_exp2**	**FC>1SD**	**FC>2SD**	**FC>3SD**	**CHROMOSOME**	**CYTOBAND**	**ENTREZ_GENE_ID**	**ILMN_GENE**
101	0.0026224	0.516318	1.1406	FALSE	FALSE	FALSE	4	4qA5	16157	IL11RA1
102	0.0266158	0.550491	1.13875	FALSE	FALSE	FALSE	3	3qF3	20226	SARS
103	0.0143911	0.538897	1.1326	FALSE	FALSE	FALSE	12	12qC3	104886	RAB15
104	0.00236728	0.516318	1.12902	FALSE	FALSE	FALSE	13	13qD2.2	19012	PPAP2A
105	0.027648	0.550491	1.11248	FALSE	FALSE	FALSE	2	2qB	74412	GLE1
106	0.00186532	0.516318	1.10599	FALSE	FALSE	FALSE	7	7qB4	20637	SNRP70
107	0.0268358	0.550491	1.104	FALSE	FALSE	FALSE	16	16qC4	114565	ZFP295
108	0.0461893	0.561001	1.08922	FALSE	FALSE	FALSE	4	4qC7	74754	DHCR24
109	0.0167724	0.538897	1.07923	FALSE	FALSE	FALSE				NR1H2
110	0.0302619	0.553513	1.07072	FALSE	FALSE	FALSE	1	1qH2.1	53330	VAMP4
111	0.0389743	0.559338	-1.04133	FALSE	FALSE	FALSE			20905	STS
112	0.0362398	0.554215	-1.04318	FALSE	FALSE	FALSE		8qB1.3	234267	GPM6A
113	0.00366052	0.516318	-1.06048	FALSE	FALSE	FALSE	11	11qB3	70510	RNF167
114	0.0335408	0.553513	-1.07456	FALSE	FALSE	FALSE			1E+08	LOC100047
115	0.0326459	0.553513	-1.08005	FALSE	FALSE	FALSE	11	11qD	629873	OTTMUSG
116	0.00604421	0.516318	-1.08521	FALSE	FALSE	FALSE			676222	LOC676222
117	0.0266307	0.550491	-1.09558	FALSE	FALSE	FALSE	15	15qF1	432982	EG432982
118	0.0318552	0.553513	-1.10538	FALSE	FALSE	FALSE	17	17qB3	63959	SLC29A1
119	0.0386245	0.55796	-1.11081	FALSE	FALSE	FALSE	9	9qF4	59289	CCBP2
120	0.0154735	0.538897	-1.12659	FALSE	FALSE	FALSE	2	2qE5	433470	AA467197
121	0.0370829	0.554215	-1.13143	FALSE	FALSE	FALSE	3	3qB	241950	BBS12
122	0.0485111	0.561001	-1.1461	FALSE	FALSE	FALSE	9	9qA4	71989	RPUSD4
123	0.0389759	0.559338	-1.14636	FALSE	FALSE	FALSE	7	7qF2	233806	TMEM159
124	0.0465533	0.561001	-1.15902	FALSE	FALSE	FALSE	11	11qC	192197	BCAS3
125	0.0129191	0.538897	-1.16747	FALSE	FALSE	FALSE		8qB3.3	234421	CIB3
126	0.0344329	0.554215	-1.17466	FALSE	FALSE	FALSE	1	1qH6	12946	CR1L
127	0.0257256	0.550491	-1.17745	FALSE	FALSE	FALSE				9330154J0
128	0.0477843	0.561001	-1.17935	FALSE	FALSE	FALSE		16qA1	66626	5730403B1
129	0.000325734	0.509961	-1.19949	FALSE	FALSE	FALSE			1E+08	LOC100044
130	0.0478787	0.561001	-1.19951	FALSE	FALSE	FALSE	15	15qB3.1	52589	NCALD
131	0.0309728	0.553513	-1.20525	FALSE	FALSE	FALSE	17	17qB1	15024	H2-T10
132	0.00146143	0.516318	-1.2179	FALSE	FALSE	FALSE	6	6qB2.3	16205	GIMAP1
133	0.0395484	0.559731	-1.21918	FALSE	FALSE	FALSE				ARID5B

	J	K	L	M	N	O	P	Q	R	S
1	**p-value(KO vs. WT)_exp2**	**FDR(p-value(KO vs. WT))_exp2**	**Fold-Change(KO vs. WT)_exp2**	**FC>1SD**	**FC>2SD**	**FC>3SD**	**CHROMOSOME**	**CYTOBAND**	**ENTREZ_GENE_ID**	**ILMN_GENE**
134	0.0271714	0.550491	-1.224	TRUE	FALSE	FALSE	2	2qF1	68126	FAHD2A
135	0.0385405	0.55796	-1.23408	TRUE	FALSE	FALSE	19	19qA	13041	CTSW
136	0.0449376	0.561001	-1.23443	TRUE	FALSE	FALSE	X	XqE3	27359	SYTL4
137	0.00278383	0.516318	-1.23875	TRUE	FALSE	FALSE	16	16qC3.1	224344	RBM11
138	0.0274346	0.550491	-1.24473	TRUE	FALSE	FALSE	16	16qB5	17470	CD200
139	0.0470799	0.561001	-1.25749	TRUE	FALSE	FALSE	6	6qE3	213393	8430408G2
140	0.0105584	0.538897	-1.26272	TRUE	FALSE	FALSE	5	5qG2	76854	GPER
141	0.0133114	0.538897	-1.28037	TRUE	FALSE	FALSE	11	11qB5	246104	RHBDL3
142	0.00753683	0.538456	-1.28441	TRUE	FALSE	FALSE	2	2qA1	74186	CCDC3
143	0.00912965	0.538897	-1.28636	TRUE	FALSE	FALSE		XqF3	53381	PRDX4
144	0.0251726	0.550491	-1.29085	TRUE	FALSE	FALSE	13	13qA3.3	18105	NQO2
145	0.0112149	0.538897	-1.29337	TRUE	FALSE	FALSE	3	3qF3	20661	SORT1
146	0.0447902	0.561001	-1.29408	TRUE	FALSE	FALSE	10	10qC1	18641	PFKL
147	0.0364949	0.554215	-1.30401	TRUE	FALSE	FALSE	9	9qC	13143	DAPK2
148	0.00485954	0.516318	-1.31344	TRUE	FALSE	FALSE	7	7qF3	11489	ADAM12
149	0.0156305	0.538897	-1.31552	TRUE	FALSE	FALSE	10	10qD2	24117	WIF1
150	0.0199814	0.538897	-1.31625	TRUE	FALSE	FALSE	6	6qF1	20315	CXCL12
151	0.000654012	0.509961	-1.32645	TRUE	FALSE	FALSE	1	1qC5	227325	DNER
152	0.0071673	0.526724	-1.34517	TRUE	FALSE	FALSE	6	6qB1	67861	2310005E1
153	0.0165897	0.538897	-1.36024	TRUE	FALSE	FALSE	16	16qC1.2	54613	ST3GAL6
154	0.0119112	0.538897	-1.38341	TRUE	FALSE	FALSE	15	15qA1	223332	RANBP3L
155	0.0469997	0.561001	-1.38892	TRUE	FALSE	FALSE	19	19qD2	14585	GFRA1
156	0.0213751	0.538897	-1.41836	TRUE	FALSE	FALSE	6	6qB3	78004	PRR15
157	0.00398404	0.516318	-1.49128	TRUE	TRUE	FALSE	11	11qB5	12569	CDK5R1
158	0.0170959	0.538897	-1.50918	TRUE	TRUE	FALSE	18	18qB3	15183	HDAC3
159	0.0456215	0.561001	-1.51065	TRUE	FALSE	FALSE	15	15qB2	69574	CMBL
160	0.0156085	0.538897	-1.51391	TRUE	TRUE	FALSE	17	17qE3	74199	VIT
161	0.00684109	0.526724	-1.52471	TRUE	FALSE	FALSE	4	4qE2	63954	RBP7
162	0.0145837	0.538897	-1.52699	TRUE	FALSE	FALSE	X	XqA3.3	54405	NDUFA1
163	0.00503672	0.516318	-1.55067	TRUE	TRUE	FALSE	3	3qA1	15213	HEY1
164	0.00624599	0.516318	-1.55183	TRUE	TRUE	FALSE	8	8qB1.1	654824	ANKRD37
165	0.00507868	0.516318	-1.59593	TRUE	TRUE	FALSE	8	8qB2	15446	HPGD
166	0.019899	0.538897	-1.59624	TRUE	TRUE	FALSE	2	2qE2	333883	CD59B

	J	K	L	M	N	O	P	Q	R	S
1	p-value(KO vs. WT)_exp2	FDR(p-value(KO vs. WT))_exp2	Fold-Change(KO vs. WT)_exp2	FC>1SD	FC>2SD	FC>3SD	CHROMOSOME	CYTOBAND	ENTREZ_GENE_ID	ILMN_GENE
167	0.036772	0.554215	-1.59796	TRUE	TRUE	FALSE	11	11qE1	15896	ICAM2
168	0.0447132	0.561001	-1.61913	TRUE	TRUE	FALSE	12	12qE	20715	SERPINA3[illegible]
169	0.0240235	0.550491	-1.72354	TRUE	TRUE	FALSE	15	15qD3	68453	GPIHBP1
170	0.00404059	0.516318	-1.72433	TRUE	TRUE	FALSE	9	9qF4	215446	ENTPD3
171	0.0257688	0.550491	-1.96729	TRUE	TRUE	FALSE	2	2qE2	12509	CD59A
172	0.0373329	0.554215	-1.96849	TRUE	FALSE	FALSE		7qF5	20287	SCT
173	0.0153481	0.538897	-2.1263	TRUE	TRUE	TRUE	5	5qE5	94111	MEPE
174	0.018448	0.538897	-2.14004	TRUE	TRUE	TRUE	12	12qA2	58185	RSAD2
175	0.0237919	0.550491	-2.17796	TRUE	TRUE	TRUE	X	XqF3	11656	ALAS2
176	0.00497677	0.516318	-2.59441	TRUE	TRUE	TRUE	1	1qD	51801	RAMP1
177	0.00278767	0.516318	-9.37215	TRUE	TRUE	TRUE	3	3qF1	20201	S100A8

	T	U	V	W	X	Y	Z	AA	AB	AC	AD
1	**SEARCH_KEY**	**SOURCE**									
2	NM_007669.2	RefSeq									
3	NM_010145.2	RefSeq									
4	scl33993.13_72	MEEBO									
5	NM_010493.2	RefSeq									
6	scl51871.1.1_203	MEEBO									
7	NM_013750.1	RefSeq									
8	NM_008737.1	RefSeq									
9	scl50457.4.570_36	RefSeq									
10	NM_053110.2	RefSeq									
11	scl33502.12_190	RefSeq									
12	scl47198.5_117	RefSeq									
13	NM_019521.1	RefSeq									
14	scl17283.6_608	RefSeq									
15	NM_028766.1	RefSeq									
16	NM_013473.2	RefSeq									
17	scl37927.8.1_330	RefSeq									
18	scl021859.7_4	RefSeq									
19	scl16393.3_24	RefSeq									
20	NM_025807.1	RefSeq									
21	NM_008184.1	RefSeq									
22	NM_153416.1	RefSeq									
23	scl35670.14_0	RefSeq									
24	NM_001033476.1	RefSeq									
25	scl30230.21.1_91	RefSeq									
26	scl37484.18.1_30	RefSeq									
27	scl20561.14_317	MEEBO									
28	NM_054077.2	RefSeq									
29	scl34770.25_118	RefSeq									
30	scl46136.14_107	RefSeq									
31	scl42068.4_0	RefSeq									
32	NM_008256.2	RefSeq									
33	NM_009128.1	RefSeq									
34	scl43651.15_178	RefSeq									

	T	U	V	W	X	Y	Z	AA	AB	AC	AD
1	**SEARCH_KEY**	**SOURCE**									
35	scl39273.6_263	RefSeq									
36	scl50042.7_106	MEEBO									
37	NM_027519.1	RefSeq									
38	NM_008780.1	RefSeq									
39	scl020310.4_115	MEEBO									
40	scl15480.1.1_277	RefSeq									
41	NM_016687.1	RefSeq									
42	NM_007907.1	RefSeq									
43	NM_009721.2	RefSeq									
44	scl43963.10.1_1	RefSeq									
45	NM_011581.1	RefSeq									
46	scl41667.16_331	RefSeq									
47	scl016010.7_252	RefSeq									
48	NM_021342.1	RefSeq									
49	NM_015734.1	RefSeq									
50	scl24434.7_205	RefSeq									
51	scl0012443.2_180	RefSeq									
52	NM_008057.2	RefSeq									
53	NM_015732.3	RefSeq									
54	scl51506.2_48	RefSeq									
55	scl0002983.1_33	RefSeq									
56	scl27971.8.1_8	RefSeq									
57	scl21044.5.1_1	RefSeq									
58	NM_007876.1	RefSeq									
59	NM_009783.1	RefSeq									
60	NM_028943.1	RefSeq									
61	NM_023699.2	RefSeq									
62	scl35378.12_17	MEEBO									
63	NM_019390.1	RefSeq									
64	NM_010786.2	RefSeq									
65	NM_010743.1	RefSeq									
66	scl46585.22_265	RefSeq									
67	scl31836.4_387	RefSeq									

	T	U	V	W	X	Y	Z	AA	AB	AC	AD
1	**SEARCH_KEY**	**SOURCE**									
68	NM_009983.2	RefSeq									
69	NM_176922.4	RefSeq									
70	NM_010638.2	RefSeq									
71	NM_021457.2	RefSeq									
72	NM_009636.1	RefSeq									
73	scl0013175.1_273	RefSeq									
74	NM_145479.2	RefSeq									
75	scl25354.23.1_78	RefSeq									
76	scl26054.30_361	RefSeq									
77	scl020338.1_17	RefSeq									
78	scl18732.66_476	RefSeq									
79	scl8260.1.1_298	RefSeq									
80	scl52445.7_23	RefSeq									
81	scl018412.1_16	RefSeq									
82	scl26588.10.546_100	RefSeq									
83	scl39780.1.1_180	RefSeq									
84	scl00208213.1_60	RefSeq									
85	NM_053168.1	RefSeq									
86	NM_013509.2	RefSeq									
87	scl51723.5_310	RefSeq									
88	scl36787.11_464	MEEBO									
89	NM_018861.2	RefSeq									
90	scl17939.35.1_294	RefSeq									
91	scl28767.1.1090_117	RefSeq									
92	scl0098730.1_284	RefSeq									
93	NM_207255.1	RefSeq									
94	NM_145476.2	RefSeq									
95	scl014104.1_1	RefSeq									
96	NM_198037.1	RefSeq									
97	scl40593.13.11_2	RefSeq									
98	scl0074029.1_132	RefSeq									
99	scl18373.4.1_231	RefSeq									
100	NM_144892.1	RefSeq									

	T	U	V	W	X	Y	Z	AA	AB	AC	AD
1	**SEARCH_KEY**	**SOURCE**									
101	NM_010549.1	RefSeq									
102	scl21649.13_184	RefSeq									
103	NM_134050.2	RefSeq									
104	NM_008903.1	RefSeq									
105	NM_028923.2	RefSeq									
106	scl000125.1_16	RefSeq									
107	scl00114565.1_269	RefSeq									
108	scl25176.1.2_129	RefSeq									
109	scl31411.7.1_30	MEEBO									
110	NM_016796.2	RefSeq									
111	NM_009293.1	RefSeq									
112	NM_153581.2	RefSeq									
113	NM_027445.1	RefSeq									
114	scl23635.5.2_0	RefSeq									
115	NM_001039502.1	RefSeq									
116	scl0016071.1_62	RefSeq									
117	NM_001034883.1	RefSeq									
118	NM_022880.1	RefSeq									
119	NM_021609.2	RefSeq									
120	NM_001004174.1	RefSeq									
121	NM_001008502.1	RefSeq									
122	scl37132.7_178	RefSeq									
123	NM_145586.1	RefSeq									
124	NM_138681.2	RefSeq									
125	scl34659.4.1_2	RefSeq									
126	scl15731.3_14	RefSeq									
127	NM_001033495.1	RefSeq									
128	NM_010443.1	RefSeq									
129	scl020341.12_22	RefSeq									
130	scl47279.22_242	RefSeq									
131	scl015039.10_13	RefSeq									
132	scl978.1.1_59	RefSeq									
133	scl37847.12_470	MEEBO									

	T	U	V	W	X	Y	Z	AA	AB	AC	AD
1	**SEARCH_KEY**	**SOURCE**									
134	scl0003028.1_68	RefSeq									
135	NM_009985.2	RefSeq									
136	NM_013757.1	RefSeq									
137	scl48967.5_235	RefSeq									
138	scl017470.1_35	RefSeq									
139	NM_145980.1	RefSeq									
140	scl27062.3_485	RefSeq									
141	NM_139228.2	RefSeq									
142	scl21321.4_587	RefSeq									
143	NM_016764.1	RefSeq									
144	NM_020282.2	RefSeq									
145	scl0020661.1_145	RefSeq									
146	NM_008826.2	RefSeq									
147	NM_010019.2	RefSeq									
148	scl000247.1_3	RefSeq									
149	NM_011915.1	RefSeq									
150	scl0001241.1_2518	RefSeq									
151	NM_152915.1	RefSeq									
152	scl067861.1_19	RefSeq									
153	scl48366.16_155	RefSeq									
154	NM_198024.1	RefSeq									
155	NM_010279.2	RefSeq									
156	NM_030024.2	RefSeq									
157	NM_009871.2	RefSeq									
158	scl51496.12_24	RefSeq									
159	NM_181588.2	RefSeq									
160	scl50486.15.1_59	RefSeq									
161	NM_022020.1	RefSeq									
162	scl54331.2.4_4	RefSeq									
163	scl22405.4_235	RefSeq									
164	scl34861.7.1_55	RefSeq									
165	scl33809.7_61	RefSeq									
166	NM_181858.1	RefSeq									

	T	U	V	W	X	Y	Z	AA	AB	AC	AD
1	**SEARCH_KEY**	**SOURCE**									
167	NM_010494.1	RefSeq									
168	scl020715.6_281	RefSeq									
169	scl068453.4_14	RefSeq									
170	NM_178676.2	RefSeq									
171	NM_007652.2	RefSeq									
172	NM_011328.1	RefSeq									
173	NM_053172.1	RefSeq									
174	NM_021384.2	RefSeq									
175	scl54562.12.1_64	RefSeq									
176	scl17654.5.1_10	RefSeq									
177	scl22940.2.1_25	RefSeq									
